# *In silico* Designing of an Epitope-Based Vaccine Against Common *E. coli* Pathotypes

**DOI:** 10.3389/fmed.2022.829467

**Published:** 2022-03-04

**Authors:** Mohamed A. Soltan, Mohammed Y. Behairy, Mennatallah S. Abdelkader, Sarah Albogami, Eman Fayad, Refaat A. Eid, Khaled M. Darwish, Sameh S. Elhady, Ahmed M. Lotfy, Muhammad Alaa Eldeen

**Affiliations:** ^1^Department of Microbiology and Immunology, Faculty of Pharmacy, Sinai University, Ismailia, Egypt; ^2^Department of Microbiology and Immunology, Faculty of Pharmacy, University of Sadat City, Sadat City, Egypt; ^3^Department of Microbiology and Immunology, Faculty of Pharmacy, Suez Canal University, Ismailia, Egypt; ^4^Department of Biotechnology, College of Science, Taif University, Taif, Saudi Arabia; ^5^Department of Pathology, College of Medicine, King Khalid University, Abha, Saudi Arabia; ^6^Department of Medicinal Chemistry, Faculty of Pharmacy, Suez Canal University, Ismailia, Egypt; ^7^Department of Natural Products, Faculty of Pharmacy, King Abdulaziz University, Jeddah, Saudi Arabia; ^8^Department of Pharmacology, Faculty of Veterinary Medicine, Zagazig University, Zagazig, Egypt; ^9^Division of Cell Biology, Histology and Genetics, Department of Zoology, Faculty of Science, Zagazig University, Zagazig, Egypt

**Keywords:** *Escherichia coli*, reverse vaccinology, immunoinformatics, epitope mapping, multitope vaccine

## Abstract

*Escherichia coli* (*E. coli*) is a Gram-negative bacterium that belongs to the family Enterobacteriaceae. While *E. coli* can stay as an innocuous resident in the digestive tract, it can cause a group of symptoms ranging from diarrhea to live threatening complications. Due to the increased rate of antibiotic resistance worldwide, the development of an effective vaccine against *E. coli* pathotypes is a major health priority. In this study, a reverse vaccinology approach along with immunoinformatics has been applied for the detection of potential antigens to develop an effective vaccine. Based on our screening of 5,155 proteins, we identified lipopolysaccharide assembly protein (LptD) and outer membrane protein assembly factor (BamA) as vaccine candidates for the current study. The conservancy of these proteins in the main *E. coli* pathotypes was assessed through BLASTp to make sure that the designed vaccine will be protective against major *E. coli* pathotypes. The multitope vaccine was constructed using cytotoxic T lymphocyte (CTL), helper T lymphocyte (HTL), and B cell lymphocyte (BCL) epitopes with suitable linkers and adjuvant. Following that, it was analyzed computationally where it was found to be antigenic, soluble, stable, and non-allergen. Additionally, the adopted docking study, as well as all-atom molecular dynamics simulation, illustrated the promising predicted affinity and free binding energy of this constructed vaccine against the human Toll-like receptor-4 (*h*TLR-4) dimeric state. In this regard, wet lab studies are required to prove the efficacy of the potential vaccine construct that demonstrated promising results through computational validation.

## Introduction

According to its pathogenicity in humans, *E. coli* can be classified into an extraintestinal infection-causing *E. coli* (primarily uropathogenic *E. coli*, UPEC, and neonatal meningitis *E. coli*, NMEC) and strains that cause enteric infections (divided into 6 pathotypes; enteropathogenic *E. coli* [EPEC], enterohemorrhagic *E. coli* [EHEC], enteroaggregative *E. coli* [EAEC], enteroinvasive *E. coli* [EIEC], enterotoxigenic *E. coli* [ETEC] and diffusely adherent *E. coli* [DAEC]). Additionally, two further pathotypes have emerged recently; adherent invasive *E. coli* [AIEC] that is predicted to be associated with Crohn's disease and does not produce any toxins, while the other pathotype, Shiga toxin-producing enteroaggregative *E. coli* [STEAEC], causes food poisoning due to the action of Shiga toxin and was reported to be the causative agent for the 2011 Germany *E. coli* outbreak (1). People can be easily infected with *E. coli* by swallowing a small amount of it in water, vegetables, or meat where the fecal-oral route is the major way of transmission (2). Another common way of categorizing *E. coli* is by serotype through detection of surface antigens O and H. Currently, more than 190 serogroups have been identified (3).

Currently, pathogenic *E. coli* is a major public health concern because of possessing a low infectious dose and simple transmission through food and water (4). Excessive usage of different classes of antibiotics, especially in cases that do not require this form of treatment, contributed largely to the appearance of antibiotic resistance in bacterial strains that were previously sensitive even for the traditional classes of antibiotics (5). Antibiotic resistance of *E. coli* is extensively studied for two major reasons; firstly, it represents the most common infective Gram-negative pathogen for humans (6), secondly, resistant *E. coli* can affect other bacteria in the gastrointestinal tract through transferring antibiotics resistance determinants (7). The resistance rates are demonstrating continuous inclement throughout the last few years (8). An alternative solution to fight against this pathogen is designing an effective vaccine against its common pathotypes. While this solution was adopted by many research groups around the world, there is no officially approved vaccine against pathogenic *E. coli* in the market (9).

Many trials have been performed to generate an effective vaccine for various *E. coli* pathotypes. Regarding intestinal *E. coli*, Shiga toxin-based vaccine (10), attenuated bacteria-based vaccine (11), and polysaccharide-based vaccine (12) have been proposed for EHEC while autotransporter-based vaccine (13) and adhesion based vaccine (14) were proposed for ETEC. Moving to extraintestinal *E. coli*, capsular-based vaccine (15) and iron scavenger receptors-based vaccine (16) were designed. The previous approaches for designing an effective vaccine are based mainly on studying the virulence properties for each pathotype of *E. coli* and as we mentioned above none of these vaccines have been approved by FDA yet. In the current study, we planned to merge two advanced approaches, reverse vaccinology and immunoinformatics for a vaccine design where these approaches would be applied on conserved protein candidates in various *E. coli* pathotypes to generate a vaccine with possible cross-reactivity.

Reverse vaccinology is a novel approach for vaccine designing that demonstrated a great development in the last few years as a result of the revolution in sequencing techniques and the data availability in highly organized databases. Additionally, computational tools that can deal with this large amount of data and translate it to valuable outcomes that can be used in applied research also showed great progress (17). This approach for vaccine design has been utilized for a protein-based vaccine design against viral, bacterial, and fungal infectious agents (18–20). Moreover, it was also applied to design an effective vaccine for animals against ETEC, a common pathotype of *E. coli* that leads to post-weaning diarrhea in pigs (21). Recent studies showed that multitope vaccines had superiority over single-component ones regarding their effectiveness (22). In the current study, we aim to investigate the whole proteome of *E. coli* and select potential protein candidates through the reverse vaccinology approach. Following that, immunogenic epitopes from these candidates would be mapped and gathered to initiate a multitope vaccine that would be assessed computationally for its, physicochemical, chemical, and immunological characteristics to be nominated as a potential vaccine candidate against pathogenic *E. coli*.

## Materials and Methods

**Figure 1:**
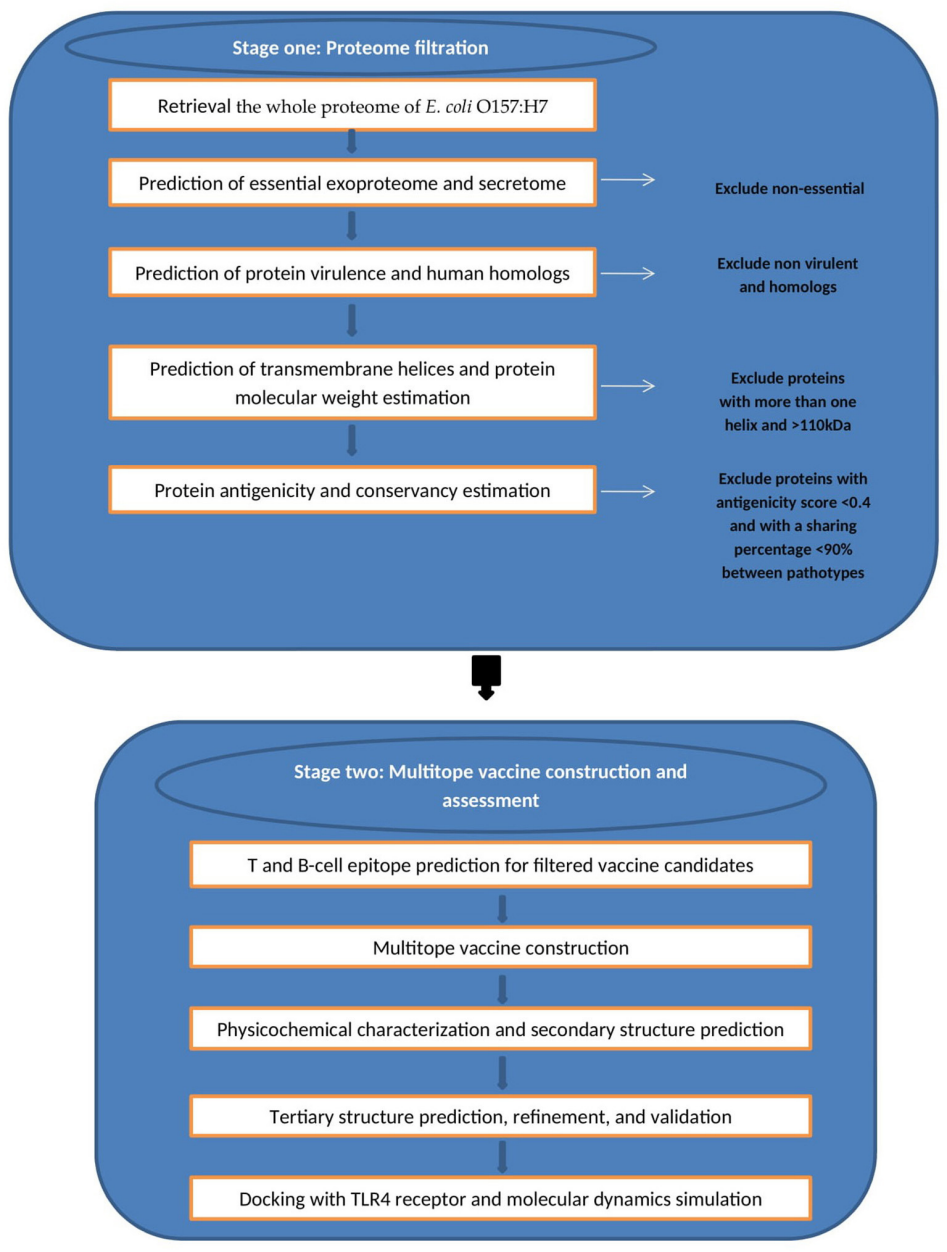
Graphical representation of the methodology employed for the multi-epitope vaccine design against *E. coli*.

We divided the current study into two main stages (Figure 1) wherein the first stage we handled the proteome of *E. coli* with successive filtration steps of reverse vaccinology approach to nominate protein candidates for vaccine design while in the second stage we constructed, analyzed, and assessed the multiepitope vaccine that was created based on B and T cell epitopes of protein candidates from the first stage.

### Data Retrieval

The amino acid sequences of the whole proteome of *E. coli* O157:H7 str. Sakai was retrieved with Genbank assembly accession No: GCF_000008865.2. This strain was selected as it represents the reference pathogenic *E. coli* strain on NCBI.

### Prediction of Essential Exoproteome and Secretome

Living organisms cannot survive without their essential genes (23). Therefore, the first filtration step was detecting these genes using Geptop 2.0, where a list of essential proteins was detected out of 5,155 proteins (the complete proteome of the studied strain) (24). The default essentiality score cutoff (0.24) was used for this process. Following that, PSORTb v3.0.2 online server (25) was employed to predict the subcellular localization of the filtered genes. The overall result of this filtration phase was a list of exoproteome and secretome essential proteins.

### Prediction of Virulence Protein

Essential exoproteome and secretome were applied to virulenpred (26) to estimate the virulence potential.

### Determination of Human Homologs

Protein list from the above stage was analyzed against human proteome on NCBI using BLASTp to check for human homologs, and those with ≥35% identity were removed from our list.

### Prediction of Transmembrane Helices and Protein Molecular Weight Estimation

The presence of transmembrane helices and proteins' molecular weight estimation were detected through TMHMM (27), and Expasy tool (28), respectively. Those with molecular weight <110 kDa and had ≤1 transmembrane helix were selected for antigenicity assessment in the next step (29).

### Protein Antigenicity Analysis

Protein Antigenicity was detected by Vaxijen V 2.0 (30), where proteins with antigenicity score more than 0.4 were considered antigenic.

### Analysis of Protein Conserved Identity With Various *E. coli* Pathotypes

Recommended proteins from previous steps were assessed for their conservation among 10 *E. coli* strains where BLASTp analysis was performed for each protein against a representative strain of each *E. coli* pathotype to confirm that the designed vaccine will have the ability to act against any pathogenic *E. coli* which is a major aim of this study. Proteins with a sharing percentage <90% were eliminated and considered not conserved.

### T-Cell and B-Cell Epitope Prediction

After completing the whole reverse vaccinology filtration steps, successful candidates were transferred to the epitope mapping stage where the filtered proteins were submitted to SignalP-5.0 Server to anticipate the location of signal peptides. Moving to the next step, the mature polypeptides were mapped for B and T cell epitopes through the online server of the Immune Epitope Database (IEBD) (31). The first epitopes to be predicted were CTLs, which were anticipated against HLA allele reference set that provided more than 97% in terms of population coverage (32). Secondly, HTLs were also predicted against HLA reference set to cover more than 99% in terms of population coverage (33). Moreover, HTL epitopes were analyzed for their ability to induce γ-INF vs. other cytokines through the webserver (http://crdd.osdd.net/raghava/ifnepitope/), IL-4 induction ability was estimated through the webserver (https://webs.iiitd.edu.in/raghava/il4pred/), while IL-10 induction ability was assessed through the webserver (https://webs.iiitd.edu.in/raghava/il10pred/). For both HTLs and CTLs, the affinity of the filtered peptides toward respective alleles was assessed where the peptide 3D structure was estimated *via* PEP FOLD 3 webserver (34); concurrently, the 3D structure of HLA-B^*^44:03 (PDB ID 4JQX) and HLA-DRB1^*^04:01 (PDB ID 5JLZ) was retrieved from the protein data bank to act as receptors for MHC-I and MHC-II epitopes, respectively, and the docking study was performed through AutoDock Vina (35). The final assessment for HTLs and CTLs was the conservancy prediction where the web-based tool from IEDB analysis resources was employed for this process (36). The last set of epitopes, BCLs were finally estimated through IEBD. After prediction, several characteristics for every single epitope were predicted to nominate the best candidates that would be used for the multitope construct. These characteristics were the population coverage, conservancy profile, antigenicity score, allergenicity, and toxicity probabilities; where IEDB analysis tools, Vaxijen, AllerTOP, and ToxinPred were employed, respectively, for these assessments.

### Multitope Vaccine Construction

In order to construct a potential multitope vaccine, the best six candidates of CTL, HTL, and BCL epitopes from the step of epitope mapping were linked through GGGS, GPGPG, and KK amino acid linkers, respectively, where these linkers were incorporated to provide *in vivo* separation of the assembled epitopes (37). Apart from the epitopes, PADRE sequence and β defensin adjuvant were added to finalize the designed vaccine construct. This potential vaccine construct was assessed for its immunogenicity score, allergenicity, and toxicity probabilities through the same servers that were employed previously for single epitopes estimation.

### Physicochemical Features, Protein Solubility Assessment, and Secondary Structure Prediction

ProtParam, a tool available on Expasy server (28) was employed to assess the physicochemical properties of the designed potential vaccine. The propensity upon overexpression in *E. coli* and the protein secondary structure of the multitope construct were anticipated *via* SOLpro server (38) and PSIPRED 4.0 webserver (39) respectively.

### Tertiary Structure Prediction, Refinement, and Validation

It is essential to predict the potential vaccine 3D structure to be able to assess its binding with a toll-like receptor. The current study utilized 3Dpro webserver (40) for this purpose. Computational estimation of a protein tertiary structure is performed through a molecule bending and twisting to create a structure with the least possible energy and maximum state of stability where the employed server runs this process *via* an analysis of the structural similarity between the protein sequence under prediction and the data available on PDB. Following tertiary structure prediction, GalaxyRefine server (41) was employed to perform refinement for the generated structure in terms of stability and protein-energy, and the enhanced structures vs. the original one were assessed by Ramachandran plot analysis (42) and ProSA (43).

### Conformational B-Cell Epitope Prediction

The conformational B-cell epitopes were analyzed for the multitope design after 3D structure prediction and refinement. The ElliPro Server (http://tools.iedb.org/ellipro), which is a reliable tool for detection of B cell epitopes against a specific antigen, was employed for this prediction (44).

### Disulfide Engineering of the Designed Vaccine

Prior to initiating a docking study for the constructed potential vaccine, it was recommended to enhance the stability of the 3D format of the designed construct. Disulfide bonds were proved to enhance protein geometric conformation and consequently elevate its stability. Disulfide by Design 2.0 (45) was assigned for this process.

### Docking of Designed Vaccine With *h*TLR-4

The current study employed molecular docking as a prediction tool for assessment of preferred orientation of the ligand, the current study vaccine construct, to its corresponding receptor and estimate the binding affinity (46). Inflammations triggered by *E. coli* are involved mainly with TLR-4 (47). Hence, the *h*TLR-4 (PDB id: 4G8A) was chosen as a receptor for the potential epitope-based constructed vaccine, the ligand, and ClusPro 2.0 server (48) was utilized to run this docking study. This server predicts the best docking models by performing billions of conformations, clustering of the 1,000 lowest energy structures generated and removing steric clashes. The 2 PDB files were uploaded to ClusPro server and the docking process was performed using default parameters.

### Dihedral Coordinate-Based Normal-Mode Analyses

The iMODS server was employed to investigate and analyze the collective flexibility as well as motion functions of the constructed epitope vaccine in relation to the bound *h*TLR-4 protein target since the latter server possesses the advantage of being fast and efficient (http://www.imods.chaconlab.org/) (49). This server can predict several values such as eigenvalues which reflected a harder deformation when this value is high (50). The atoms and residues of both the bound *h*TLR-4 protein and epitope vaccine ligand were continuously indexed where atoms of number range 1–9,567 and 9,568–15,225 were, respectively, assigned for the epitope-bound *h*TLR-4 (the initial 27–627 amino acids) and epitope vaccine itself (the following 1–380 amino acids).

### Molecular Dynamics Simulations

The molecular dynamics computational approach was applied to describe the epitope/*h*TRL-4 molecular behaviors in addition to measuring the stability of such protein-protein complex (51). The *h*TLR-4 can exist in a monomeric state as well as m-shaped dimeric architecture where the latter is prior to the initiation of downstream signal transduction (52, 53). Additionally, reported crystalline *h*TLR-4 showed a significant *N*-glycosylation profile with several oligosaccharide moieties being linked to their surface at conserved residues (54, 55). In these regards, four docked *h*TLR-4/epitope vaccine complexes were investigated for the impact of glycosylation and/or *h*TLR-4 oligomerization state on the binding affinity as well as the thermodynamic stability of the constructed epitope vaccine. Simulated complexes were dimeric or monomeric *h*TLR-4 states being either glycosylated or not (Glycosylated dimer = GlyDim; Sole dimer = SolDim; Glycosylated monomer = GlyMon, Sole monomer = SolMon). Each of the latter adopted models were individually subjected to 100 ns explicit molecular dynamics runs using GROMACS-2019 (http://www.gromacs.org/) (56) and under CHARMM36m forcefield for the protein simulations (57–60). Under periodic boundary conditions, the TIP3P water model 3D-box was used to solvate the investigated protein-protein complex model with 10 Å marginal distances (61). The standard ionization state of both protein amino acids was assigned under physiological pH 7.4, whereas the whole constructed system charge was *via* sufficient chloride and potassium ion numbers introduced through Monte-Carlo ion-placement method (62). The atomic counts of the four constructed models were 438642, 424612, 242818, and 237898 atoms for GlyDim; SolDim; GlyMon, and SolMon, respectively.

The constructed system was minimized throughout 5 ps using a steepest-descent algorithm (63), and subsequently subjected to double-staged equilibration for 100 ps/stage under a constant number of particles, Volume, and Temperature (NVT; 303.15 K, Berendsen temperature coupling regulation) and a constant number of particles, Pressure, and Temperature (NPT; 303.15 K and 1 atm. Pressure, Parrinello-Rahman barostat regulation) ensembles for the first and second stage, respectively. Throughout both minimization and equilibration stages, the original protein foldings were preserved and all heavy atoms were restrained at 1,000 kJ/mol.nm^2^ force constant. Finally, the minimized/equilibrated systems were produced through 100 ns explicit molecular dynamics runs under NPT ensemble using Particle-Mesh Ewald algorithm for long-range electrostatic interactions computation. Linear constraint LINCS method modeled the covalent bond lengths at 2 fs integration time step sizes (64). van der Waals and Coulomb's non-bounded interactions were truncated at 10 Å *via* Verlet cut-off schemes (65). The MD simulations were performed using Aziz® Supercomputer (King Abdulaziz University's High-Performance Computing Center), *via* 5 nodes with 24 CPUs/node and 8 MPI processes/node. Adopted nodes run CentOS-6.4 with dual Intel® E5-2695v2 (24 cores/node; i.e. 2.4 GHz 12 Cores) offering 96 GB Memory/node. The MD simulation of the dimer *h*TLR-4 states took ~228 and 218 h for the glycosylated and non-glycosylated proteins, respectively. While as the monomeric states took nearly 132 and 128 h for the glycosylated and non-glycosylated proteins, respectively.

The trajectory-oriented analytical parameters; root-mean-square deviation (RMSD), RMS-fluctuation (RMSF), radius of gyration (Rg), and solvent-accessible surface area (SASA) were computed through molecular dynamics trajectory analysis using GROMACS built-in analytical scripts. The free binding energies, as well as residue-wise energy contribution between *h*TLR-4 protein target and epitope vaccine ligand, were estimated *via* Molecular Mechanics/Poisson-Boltzmann Surface Area (MM/PBSA) using GROMACS/*g_mmpbsa* scripts (66). The SASA-only model of the free-binding energy (Δ*G*_*Total*_ = Δ*G*_MolecularMechanics_ + Δ*G*_*Polar*_ + Δ*G*_*Apolar*_) was used across the whole 100 ns molecular dynamics runs. Important MM/PBSA parameters for polar/solvation calculations were set at 1.40 Å solvent probe radius, 80 pdie solvent dielectric constants, 1 vdie standard vacuume, and 2 pdie solute dielectric constants. Regarding only-SASA non-polar solvation; 1.40 Å SASA solvent probe radius, 0.0227 kJ/mol.Å^2^ solvent surface tension, and 3.8493 kJ/mol offset constant, were used. Finally, parameters for the continuum-integral-based model were set as 1.25 Å solvent probe radius, 200 quadrature points/Å^2^, and 0.0334 Å^3^ bulk solvent density. Representing ligand-protein conformations at specific timeframes was done *via* Schrödinger-Pymol V.2.0.6 graphical package.

### Immune Simulation of the Designed Vaccine

The C-ImmSim server (67) was employed to predict the stimulated immune response against the designed vaccine through a computational approach. We followed the technique of prime—booster—booster for this investigation and that was achieved by injecting the designed vaccine three times with 4 weeks intervals. This approach was applied to obtain a long-lasting immune response.

## Results

**Figure 2:**
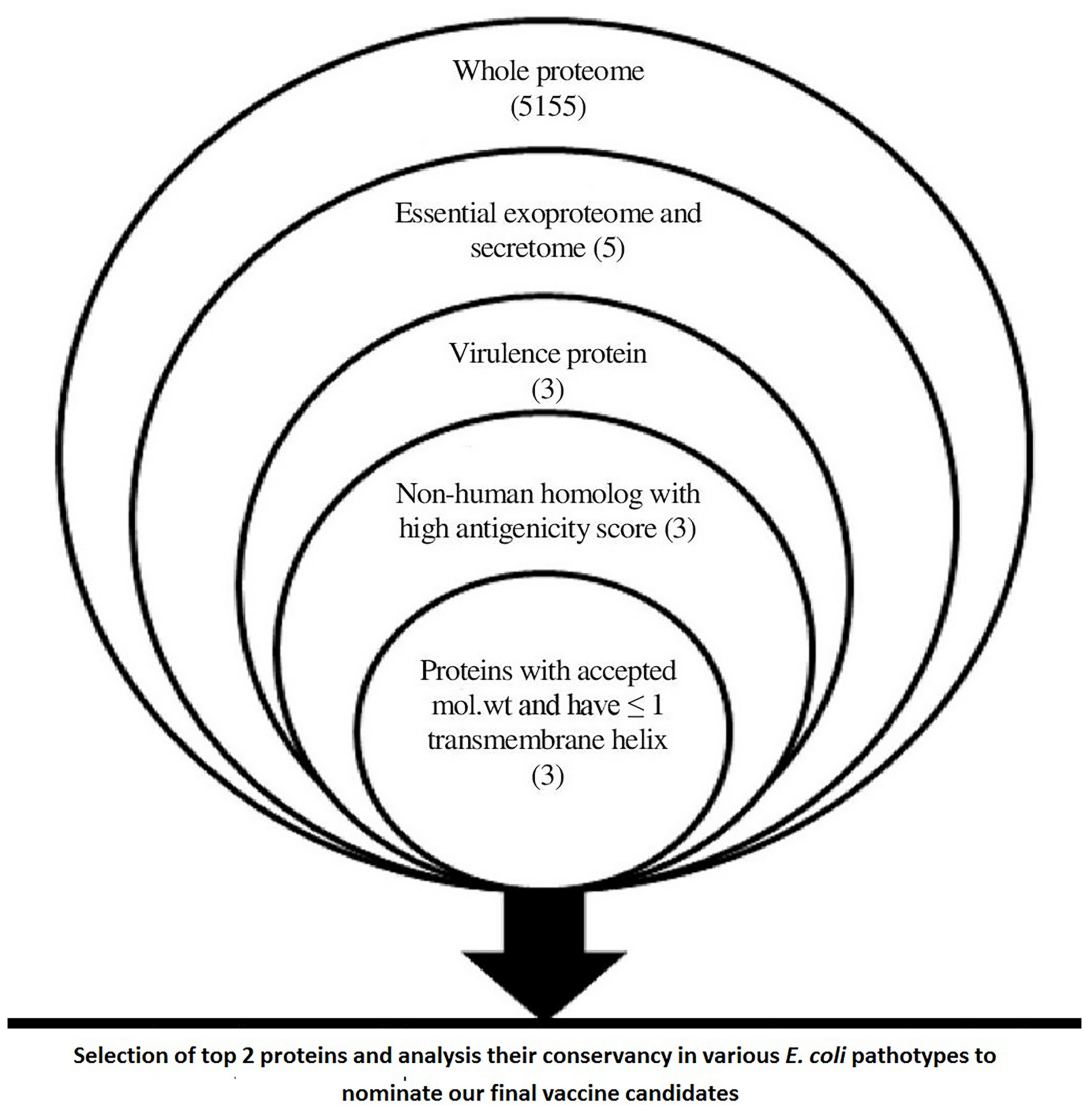
A chart summarizing applied filtration steps for the nomination of potential vaccine candidates by applying *in silico* reverse vaccinology technique.

The main output of the filtration steps that have been applied in the current study to define potential candidates are shown in Figure 2.

### Proteome Analysis for Selection of Vaccine Candidates

Data within Figure 2 demonstrates the results of filtration steps of *E. coli* O157:H7 str. Sakai proteome where three proteins (lipopolysaccharide assembly protein LptD, outer membrane protein assembly factor BamA, and lipopolysaccharide assembly protein LptE) matched all the requirements. We selected the top 2 proteins that passed all the filtration steps and had the highest antigenicity score to obtain an accepted length of multiepitope vaccine after connecting the best-predicted T and B cell epitopes from these 2 proteins so we nominated LptD and BamA as our final vaccine candidates, characteristics of these candidates are shown in Supplementary Table 1.

### Conservation Analysis of Nominated Vaccine Candidates Among *E. coli* Pathotypes

Conservation analysis of our nominated vaccine candidates showed that these proteins were found with high conservation among various *E. coli* pathotypes (Supplementary Table 2) which confirmed their ability to protect against different *E. coli* infections, therefore we designed the multitope vaccine based on these 2 proteins.

### T Cell Epitopes

In order to select the best T cell epitopes for constructing the multitope vaccine, the top 100 generated peptides from IEBD per each protein candidate were estimated for their antigenicity score, allergenicity, and toxicity probabilities, and the top 10 peptides, which demonstrated the lowest percentile rank, the highest binding affinity and antigenicity score more than 0.4 were grouped in Table 1 (for MHC-I peptides) and Table 2 (for MHC-II peptides). The binding affinity of the selected candidates of CTLs and HTLs was assessed through a docking study where the generated docked complexes for CTLs are shown in Supplementary Figure 1 and the docked complexes for HTLs are shown in Supplementary Figure 2. The binding scores of all complexes are shown in Supplementary Table 3. These scores ranged between −7.6 and −8.5 for CTLs, and from −7.1 to −8.1 for HTLs. The receptors of this docking study were deposited in the protein data bank with docked peptides that were firstly removed before running the current docking analysis then docked again separately to their respective receptor to act as a control for our docking study. The binding score for MHC-I control was −7.5 and that of MHC-II was −7.6, By comparing the binding energy score for our candidate list and the controls we can confirm that these candidates are promising to be selected for the construction of the multitope vaccine. Another significant factor that was considered in the selection of the epitopes for constructing the multitope vaccine was the population coverage. The IEDB population coverage analysis tool was employed for this process. The whole list of predicted epitopes for both protein candidates showed the epitopes arranged in descending way based on their binding affinity to the different alleles therefore we selected the top 10%, that would represent the epitopes with high binding affinity to respective alleles, and collected these alleles per each epitope to analyze the population coverage for single epitopes (Supplementary Table 4) then we analyzed the population coverage for the combined CTLs, combined HTLs and the multitope vaccine (Supplementary Table 5).

**Table 1:** Top-ranked T-cell epitopes (MHC-I peptides) of BamA and LptD proteins.

**Epitope**	**Protein**	**Antigenicity**	**Allergenicity**	**Toxicity**	**Immunogenicity**	**Conservancy (%)**
KTDDFTFNY	BamA	1.74	Non	Non	0.2750	100
FKTDDFTFNY	BamA	1.54	Non	Non	0.3020	100
MSAGIALQW	BamA	1.41	Non	Non	0.1361	100
NVDAGNRFY	BamA	0.41	Allergen	Non	0.1855	100
AELSVTNPY	BamA	0.97	Non	Non	−0.1052	100
AEIQQINIV	BamA	0.78	Non	Non	0.0114	100
YANSVRTSF	BamA	0.53	Allergen	Non	−0.1435	100
RMSAGIALQW	BamA	1.5	Allergen	Non	0.1080	100
QADDADLSDY	BamA	0.82	Non	Non	−0.0478	100
YSDPSNIRM	BamA	0.82	Non	Non	−0.0285	100
RTGDDNITW	LptD	1.7	Allergen	Non	0.1838	100
FSEQNTSSY	LptD	0.43	Non	Non	−0.2901	100
KLDESVNRV	LptD	0.79	Non	Non	0.0104	100
RIYGQAVHF	LptD	0.66	Non	Non	0.0106	100
SPEYIQATL	LptD	0.49	Allergen	Non	0.1050	100
ATSNSSIEY	LptD	1.13	Allergen	Non	−0.2062	100
KVGPVSIFY	LptD	0.71	Non	Non	0.0650	100
TLEPRAQYLY	LptD	1.04	Non	Non	−0.0088	100
IYDDAAVERF	LptD	0.5	Allergen	Non	0.2593	100
KQADSMLGV	LptD	0.8	Allergen	Non	−0.2809	100

**Table 2:** Top-ranked T-cell epitopes (MHC-II peptides) of BamA and LptD proteins.

**Epitope**	**Protein**	**Antigenicity**	**Allergenicity**	**Toxicity**	**INF-γ**	**IL-4**	**IL-10**	**Conservancy (%)**
DPSNIRMSAGIALQW	BamA	1.26	Non-allergenic	Non-toxic	Positive	Inducer	Non-inducer	100
PSNIRMSAGIALQWM	BamA	1.22	Non-allergenic	Non-toxic	Positive	Non-inducer	Non-inducer	100
SNIRMSAGIALQWMS	BamA	1.03	Non-allergenic	Non-toxic	Positive	Non-inducer	Non-inducer	100
KLAGDLETLRSYYLD	BamA	0.66	Allergenic	Non-toxic	Positive	Inducer	Non-inducer	100
QKLAGDLETLRSYYL	BamA	0.62	Allergenic	Non-toxic	Positive	Inducer	Non-inducer	100
NIRMSAGIALQWMSP	BamA	1.25	Non-allergenic	Non-toxic	Positive	Non-inducer	Non-inducer	100
QRVAVGAALLSMPVR	BamA	0.51	Non-allergenic	Non-toxic	Positive	Non-inducer	Non-inducer	100
DYTNKSYGTDVTLGF	BamA	0.79	Allergenic	Non-toxic	Positive	Inducer	Non-inducer	100
NKSYGTDVTLGFPIN	BamA	0.99	Non-allergenic	Non-toxic	Positive	Inducer	Non-inducer	100
TNKSYGTDVTLGFPI	BamA	1.07	Non-allergenic	Non-toxic	Positive	Inducer	Non-inducer	100
GPVSIFYSPYLQLPV	LptD	0.68	Non-allergenic	Non-toxic	Positive	Non-inducer	Non-inducer	100
VQLNYRYASPEYIQA	LptD	1.11	Allergenic	Non-toxic	Positive	Inducer	Non-inducer	100
VGPVSIFYSPYLQLP	LptD	0.69	Allergenic	Non-toxic	Positive	Inducer	Inducer	100
VSIFYSPYLQLPVGD	LptD	0.45	Allergenic	Non-toxic	Positive	Inducer	Inducer	100
LNYRYASPEYIQATL	LptD	0.88	Allergenic	Non-toxic	Positive	Inducer	Non-inducer	100
KVGPVSIFYSPYLQL	LptD	0.88	Non-allergenic	Non-toxic	Positive	Inducer	Inducer	100
NYRYASPEYIQATLP	LptD	0.66	Allergenic	Non-toxic	Positive	Inducer	Non-inducer	100
YLPYYWNIAPNMDAT	LptD	1.76	Allergenic	Non-toxic	Positive	Inducer	Inducer	100
AKYTTTNYFEFYLPY	LptD	1.13	Non-allergenic	Non-toxic	Positive	Inducer	Non-inducer	100
SSIEYRRDEDRLVQL	LptD	0.81	Non-allergenic	Non-toxic	Positive	Non-inducer	Inducer	100

### B-Cell Epitope Identification

Bepipred Linear Epitope Prediction 2.0 was used as a prediction method. It identified 30 and 21 B-cell epitopes for BamA and LptD proteins, respectively. Peptides with a length between 9:18 amino acids were analyzed for their antigenicity and peptides with antigenicity score >0.4 were tested for their allergenicity and toxicity (Table 3).

**Table 3:** Predicted B-cell epitopes of BamA and LptD proteins.

**BamA**				**LptD**			
**Epitope**	**Antigenicity**	**Allerge-nicity**	**Toxicity**	**Epitope**	**Antigenicity**	**Allerge-nicity**	**Toxicity**
PVRTGDTVNDEDIS	1.24	Allergen	Non	KEAPGQPEPV	0.96	Non	Non
ASGVRVGESLDRT	0.97	Non	Non	DKVYEDEHPNDDSS	0.41	Non	Non
IRFEGNDTSKDAV	1.14	Non	Non	PSYFNDFDNKYGSSTDGY	1.22	Non	Non
TDTQRVPGSP	0.49	Non	Non	QVFSEQNTSSYS	0.42	Non	Non
FQADDADLSDYTNK	0.56	Non	Non	QTNLDWYNSRNTTKLDES	0.75	Non	Non

### Construction of Multitope Vaccine

From Tables 1–3, six epitopes per each table (three from each protein candidate) were chosen based to constitute the basis of the multitope vaccine (graphical representation for the constructive components is shown in Supplementary Figure 3). Moreover, β-defensin and PADRE peptide were also incorporated to finalize a potential vaccine sequence of 380 amino acids in length and its sequence was as the following:

“EAAAKGIINTLQKYYCRVRGGRCAVLSCLPKEEQIGKCSTRGRKCCRRKKEAAAKAKFVAAWTLKAAAGGGSKTDDFTFNYGGGSAELSVTNPYGGGSAEIQQINIVGGGSFSEQNTSSYGGGSRIYGQAVHFGGGSTLEPRAQYLYGPGPGDPSNIRMSAGIALQWGPGPGQRVAVGAALLSMPVRGPGPGTNKSYGTDVTLGFPIGPGPGKVGPVSIFYSPYLQLGPGPGAKYTTTNYFEFYLPYGPGPGSSIEYRRDEDRLVQLKKASGVRVGESLDRTKKIRFEGNDTSKDAVKKTDTQRVPGSPKKKEAPGQPEPVKKPSYFNDFDNKYGSSTDGYKKQTNLDWYNSRNTTKLDESKKAKFVAAWTLKAAAGGGS”.

This construct was assessed to be non-allergen with an antigenicity score of 1.07 (estimated by VaxiJen v2.0) and 0.959 (estimated by ANTIGENpro).

### Physicochemical Properties Assessment and Secondary Structure Prediction

**Figure 3:**
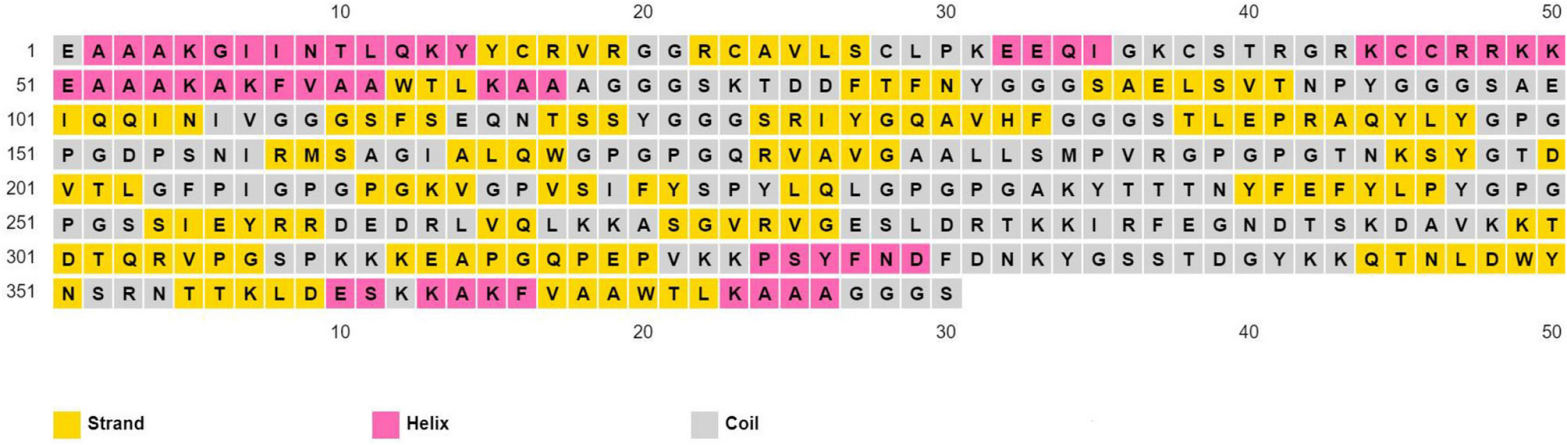
Secondary structure prediction of designed multitope vaccine using PESIPRED server.

The physicochemical properties of the predicted vaccine construct were detected by using the ProtParam server and demonstrated in Supplementary Table 6. The designed vaccine had a SOLpro SVM score of 0.95; therefore it was predicted to be soluble as SOLpro values > 0.5 are considered as soluble. Vaccine secondary structure prediction demonstrated the presence of 14.2% helix, 37.9% strand, and 47.9% coil structure (Figure 3).

### Tertiary Structure Prediction, Refinement, and Validation

**Figure 4:**
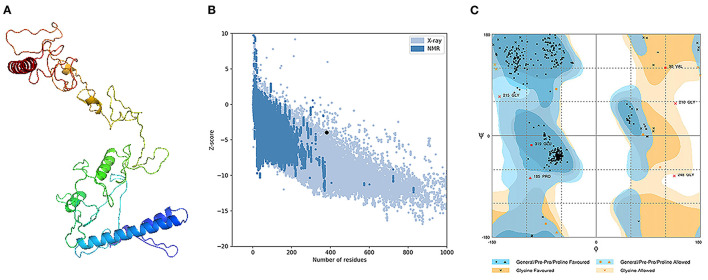
Structural analysis of the predicted 3D structure of the vaccine. **(A)** The three-dimensional structure of vaccine obtained after molecular refinements; **(B)** ProSA-web evaluation of the vaccine structure; **(C)** Ramachandran plot analysis of the protein structure after molecular refinements.

The assessment of the primary 3D structure, through Ramachandran plot analysis and ProSA online server, demonstrated that 87.6, 6.3, and 6.1% of residues were located in favored, allowed, and outlier regions, and the *Z*-score −3.45, respectively. While these values may be considered acceptable for a predicted 3D structure, we continued with refinement for better structure creation. Protein refinement occurred through the help of GalaxyRefine web server and the best model, regarding scores improvement, Figure 4A demonstrated the following scores' enhancement, the Z-score enhanced from −3.45 to −3.9 (Figure 4B) while the Ramachandran plot analysis scores became 96%, 2.4%, and 1.6% for residues in favored, allowed, and outlier regions, respectively (Figure 4C).

### Conformational B-Cell Epitope Prediction

The tertiary structure and folding of the designed vaccine may generate new conformational B-cell epitopes and for this purpose, we used ElliPro server conformational. In the current assessment, the server predicts 9 new epitopes and their scores were between 0.514 and 0.84 (Supplementary Table 7). The predicted 3D models of the generated epitopes are shown in Supplementary Figure 4.

### Vaccine Disulfide Engineering

Usage of DbD2 server for disulfide bond assign demonstrated that 26 pairs of amino acids are eligible to make disulfide bonds while in terms of other parameters such as energy and Chi3 value, this number reduced to only 2 pairs. Therefore, 4 mutations were performed at the residues pairs of SER89-ARG187 and PRO324-PHE330. The followed values of energy and Chi3 to recommend disulfide engineering were below 2.2 and from −87 to +97, respectively.

### Molecular Docking of the Vaccine With TLR4

**Figure 5:**
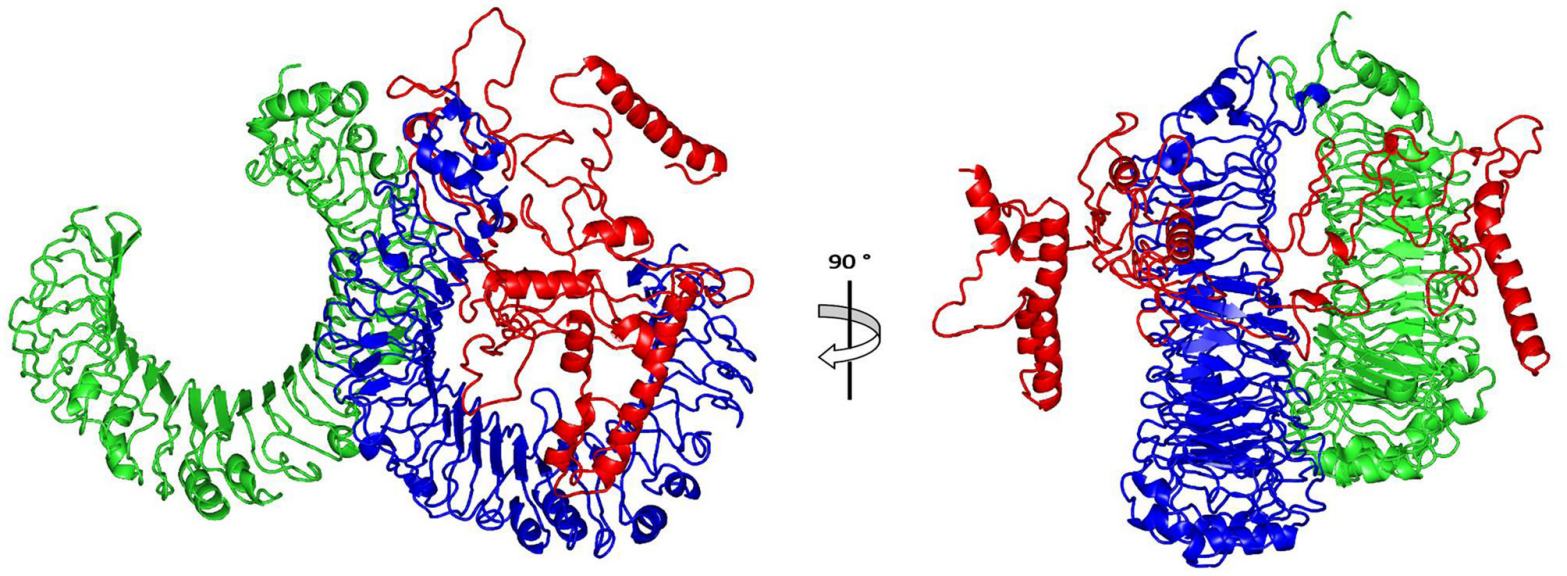
Docked complex of vaccine constructs with human TLR4; vaccine constructs in red color and TLR4 receptor in green and blue colors.

The ClusPro 2.0, which was employed for the docking study, generated 30 models and the model number 0.00 (Figure 5) exhibited the lowest binding energy score of −1420.9 kcal/mol which implicate a good affinity and stability of the constructed complex.

### Dihedral Coordinate-Based Normal-Mode Analyses

**Figure 6:**
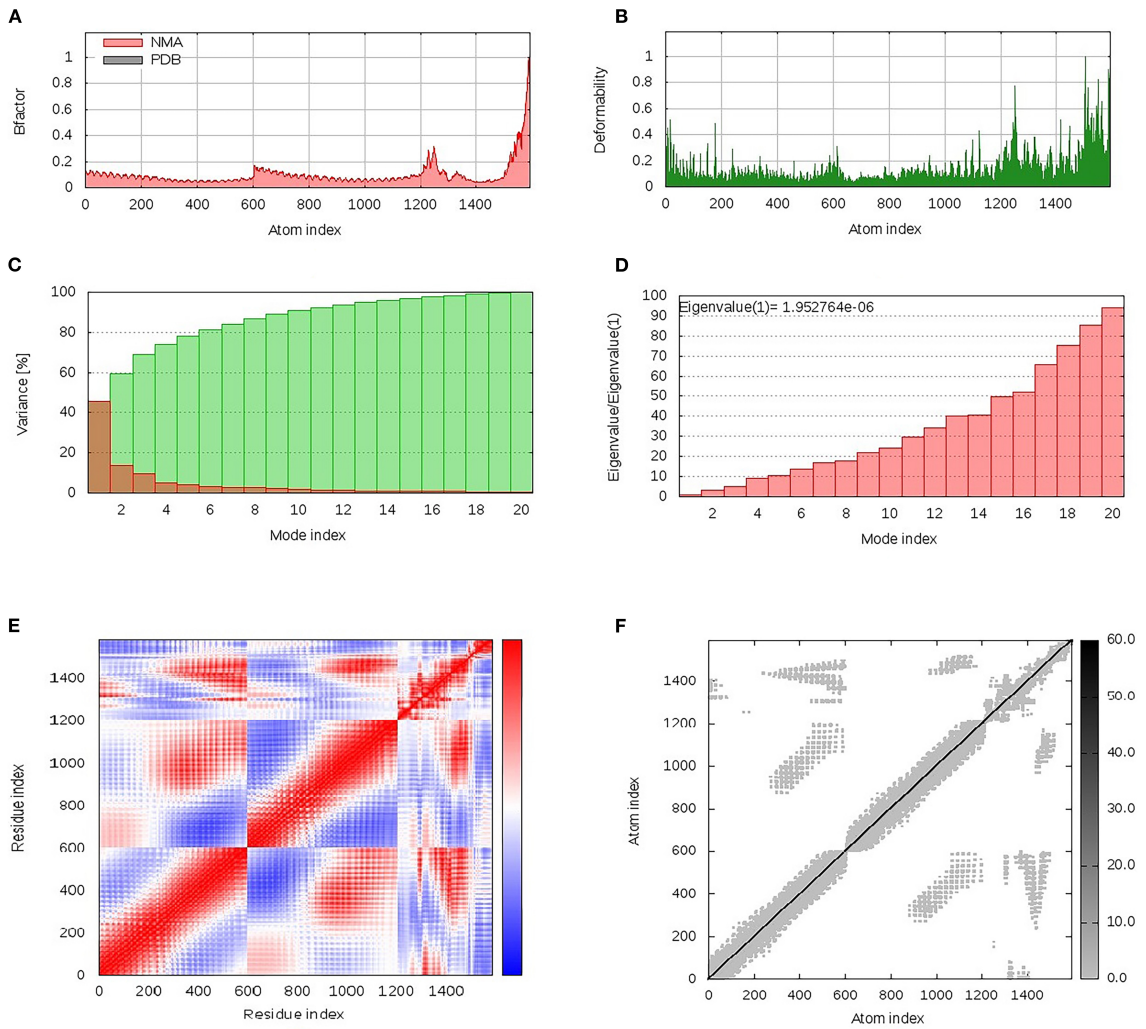
Dihedral coordinate-based normal-mode analyses of multitope vaccine-*h*TLR-4 complex; ligand-receptor interaction was assessed throughout comparative **(A)** B-factor indices, **(B)** deformabilities, **(C)** variance, **(D)** eigenvalues, **(E)** covariance of residue indices, and **(F)** elastic network analysis.

Within the iMOS server finding analysis, B-factor correlates with the relative magnitude of atom displacement around conformational equilibria. Values were significantly higher for the epitope vaccine ligand (atom index; 9,568–15,225), particularly at its respective carboxy terminus, in relation to that of the *h*TLR-4 target protein (atom index; 1–9,567) (Figure 6A). The B-factor results were recapitulated *via* the complex deformability index presented in Figure 6B where each vaccine residue, particularly at the carboxy end, showed individual distortions being higher than those of the *h*TLR-4 target protein. The estimated eigenvalue, which represents the motion stiffness of the complex, was 1.95e^−06^, where being in inverse order in relation to variance predicting the significantly higher mobility of the vaccine as compared to the *h*TLR-2 complex across collective functional motions (Figures 6C,D). The iMOS provided the covariance matrix illustrating the coupled residue pairs demonstrating anti-correlated (blue color), correlated (red color), or uncorrelated (white color) motions. The *h*TLR-4 depicted lower predicted correlated residue-pair motions than did the epitope vaccine ligand, however, the latter protein showed less anti-correlated motions (Figure 6E). Finally, the obtained elastic-network model explains the differential flexibility patterns among both investigated proteins (Figure 6F). Represented in different colors, the elastic-network model describes the atom pairs linked *via* springs relying on the stiffness degree between them. Stiffer strings were correlated to dark gray colors. The target *h*TLR-4 protein showed continuous dark-gray bands along the normal distribution of stiffer string, while the residues of the epitope vaccine illustrated non-continuous gray bands around the same immobility normal string, particularly for those residues near the carboxy terminus.

### Molecular Dynamics Simulation Analyses

**Figure 7:**
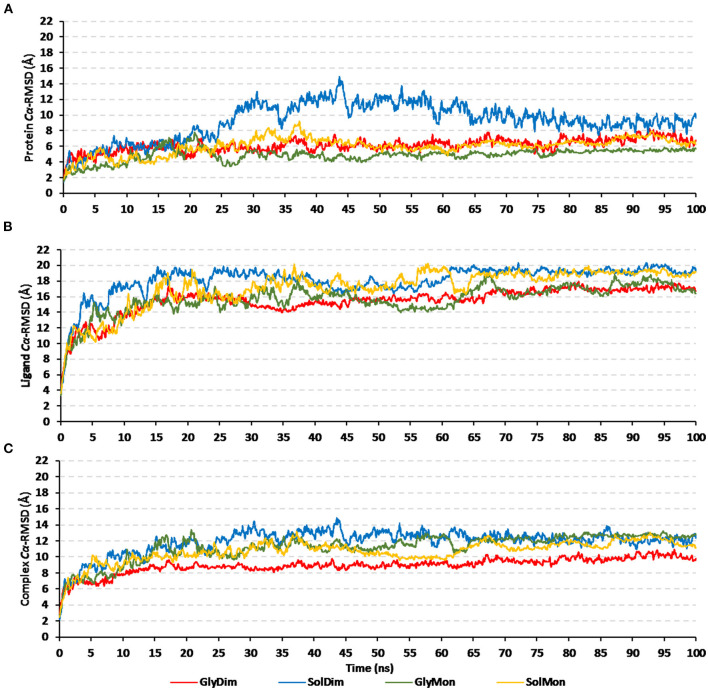
RMSD trajectory analysis for the four investigated epitope vaccine ligand-bound *h*TLR-4 target models across 100 ns explicit molecular dynamics simulation runs. Time-evolution of **(A)** Proteins' *C*α-RMSD, **(B)** Ligands' *C*α-RMSD, and **(C)** Complexes' *C*α-RMSD, along molecular dynamics timeframes (ns).

Throughout the 100 ns molecular dynamics runs, each simulated protein depicted thermodynamic behavior being typical through molecular dynamics simulations (Figure 7). The monitored proteins' RMSD deviations, in relation to corresponding alpha-carbon (*C*α-RMSD) of the reference protein, showed an initial increase over the initial frames owing to the release of constraints at the beginning of the simulation stage. Steady RMSD tones were depicted for almost all protein models beyond the first 30 ns and till the end of the molecular dynamics runs (i.e., for >75 ns). The RMSD trajectories for the epitope vaccine and respective bound hTLR-4 were around two-fold differences the thing that ensured sufficient protein convergence as well as significant ligand accommodation at the target pocket. The latter findings ensured the adequacy of the 100 ns MD simulation timeline to grasp sufficient thermodynamic information within efficient computational cost and without the need for more time extension. Regarding comparative *C*α-RMSDs analysis between the *h*TLR-4 target proteins and their corresponding in-bound epitope vaccine ligands, higher RMSD trajectories were illustrated for the ligand proteins (Figures 7A,B). The latter was obvious since the obtained average *C*α-RMSD values across the protein's trajectory plateau and till MD simulation end courses were 8.58 ± 0.72 and 15.92 ± 0.32 Å for the *h*TLR-4 and vaccine ligands, respectively. Concerning the comparative RMSD analysis for the oligomeric *h*TLR-4 states, steadier and lower *C*α-RMSD fluctuation were depicted for the dimeric state as compared to those of the monomer ones. On similar bases, significantly lower and more steady *C*α-RMSD tones were depicted within models where the *h*TLR-4 target proteins were covalently bound to higher oligosaccharides. Except for limited fluctuations, the monitored *C*α-RMSDs of the combined *h*TLR-4/epitope proteins showed rapid equilibration plateaus (11.23 ± 0.11 Å), beyond 25 ns and till the end of the simulations runs (Figure 7C). Notably, the least fluctuating complex *C*α-RMSD trajectories (9.30 ± 0.57 Å) were seen for the dimeric *N*-glycosylated *h*TLR-4/vaccine system.

**Figure 8:**
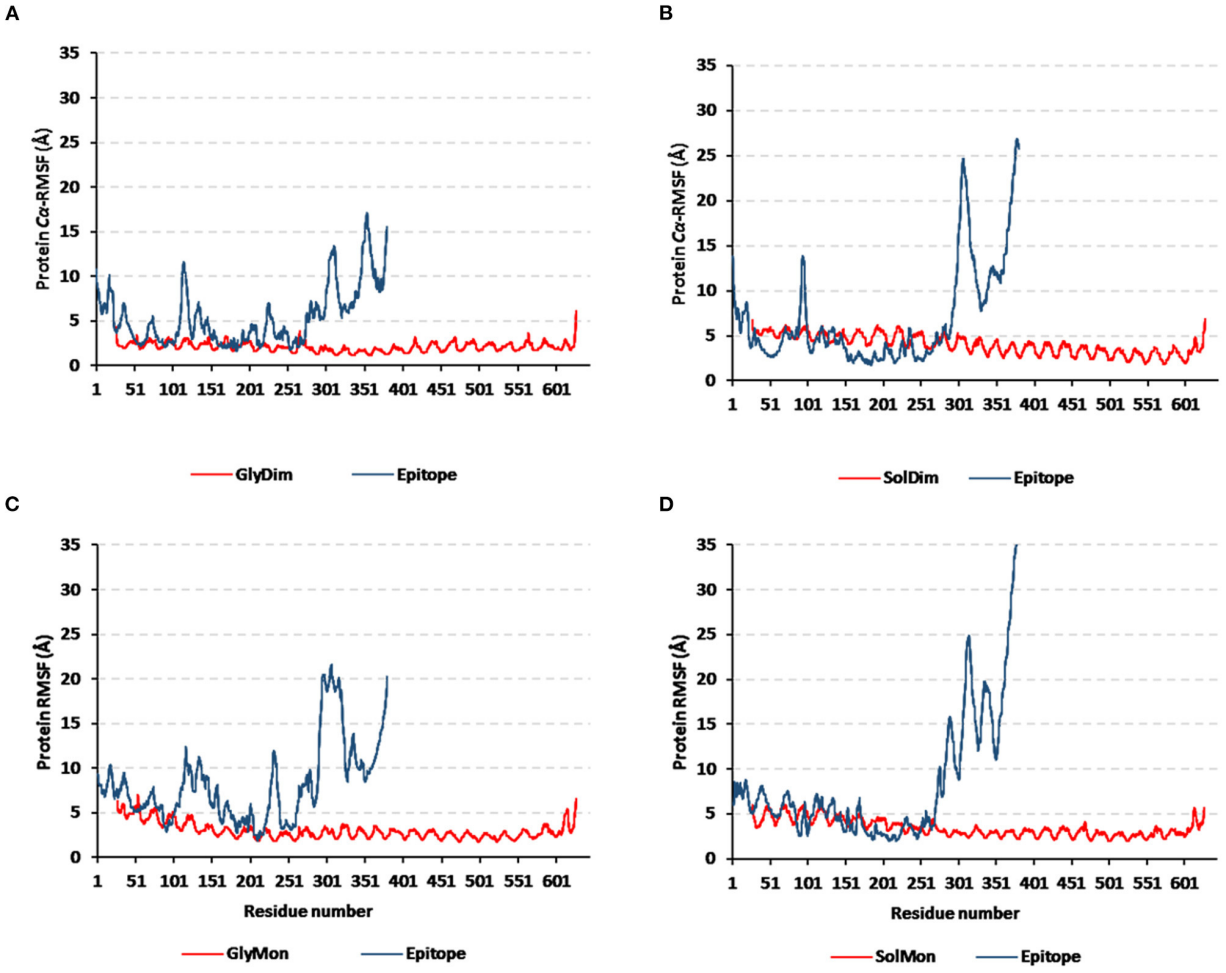
Protein's residue-wise fluctuation patterns. Monitored *C*α-RMSF tones across the whole 100 ns molecular dynamics runs for the investigated hTLR-4 bound epitope vaccine within different oligomeric/saccharide-linked states. **(A)** glycosylated dimeric, **(B)** non-glycosylated dimeric, **(C)** glycosylated monomeric, and **(D)** non-glycosylated monomeric states, along the sequence residue numbers (bound TLR-4 protein target = 27–627; epitope vaccine = 1–380 residue ranges).

Monitoring the *C*α-RMSF tones across the whole simulated trajectories (100 ns) provided interesting information regarding the residue-wise fluctuation pattern of each corresponding simulated protein. Interestingly, typical well-behaved molecular simulation profiles were depicted for each simulated protein where terminal residues and their vicinal ranges showed higher mobility patterns (high *C*α-RMSF) as compared to those at the core regions (Figure 8). As a general observation, the epitope vaccine ligands showed much higher residue-wise fluctuation profiles as compared to their corresponding in-bound *h*TLR-4 target proteins. The latter observation was most recognized for the vaccines' respective carboxy end amino acids as well as their vicinal residues (high residue sequence numbering; from 301 to 380) in relation to those of the *N*-terminus. Notably, the depicted vaccine-oriented fluctuation trends were more associated with monomeric/non-*N*-glycosylated *h*TLR-4 state (*C*α-RMSF up to 35 Å) as compared to other oligomeric models. On similar bases, the lowest RMSF-based mobility trends were assigned to the *N*-glycosylated dimeric *h*TLR-4 model as compared to any other simulated model.

**Figure 9:**
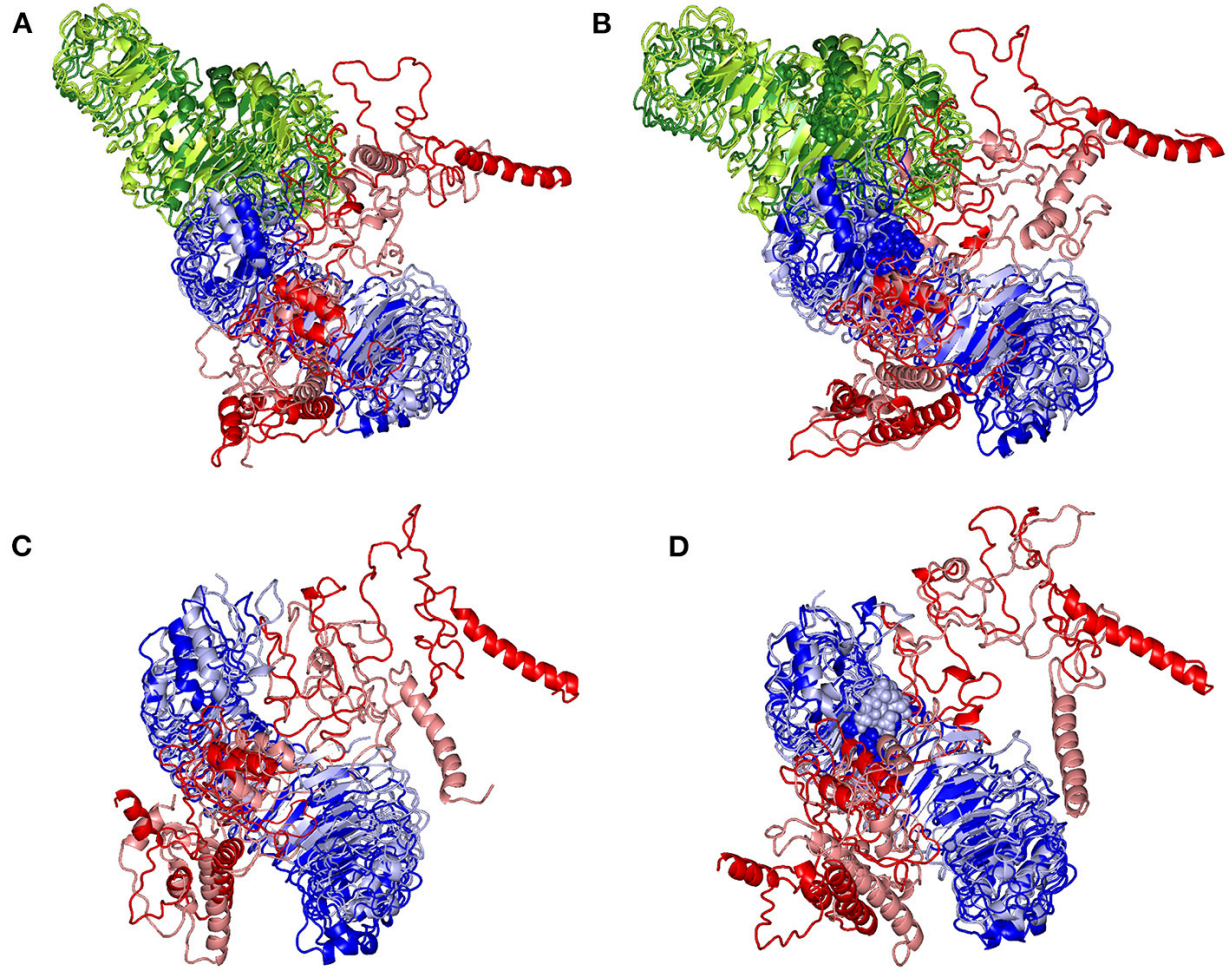
Conformational changes for the simulated *h*TLR-4/epitope vaccine complexes. Overlaid snapshots of the 3D-cartoon representation of the; **(A)** glycosylated dimeric, **(B)** non-glycosylated dimeric, **(C)** glycosylated monomeric, and **(D)** non-glycosylated monomeric states at 0 ns and 100 ns of the molecular dynamics runs. The *h*TLR-4 target proteins (blue/green) and epitope vaccine ligands (red) are colored in respective to the initial or last extracted frames (dark and light colors are for 0 ns and 100 ns extracted frames, respectively). Moieties of *N*-glycosylation are presented as spheres and colored in respective to their linked *h*TLR-4 protomer and extracted frame.

Subsequent analysis of the key alterations within the conformations of both simulated epitope vaccine and *h*TLR-4 protein was proceeded throughout examining each simulated model at the initial and last molecular dynamics timeframes. Extracted frame lines at 0 and 100 ns were subjected to 1 × 10^−3^ Kcal/mol.A^2^ gradient minimization using MOE-Molecular Operating Environment software. The RMSDs of the overlaid conformations were 6.645, 9.855, 5.488, and 7.784 Å for the glycosylated dimeric, non-glycosylated dimeric, glycosylated monomeric, and non-glycosylated monomeric complexes. Notably, all simulated models illustrated stable binding states for the epitope vaccine at the *h*TLR-4 binding site (Figure 9). Limited conformational changes were assigned for the *h*TLR-4 target protein across the four simulated models. On the contrarily, the simulated epitope vaccines depicted significant conformational changes causing them to adopt a more compacted conformation/orientation at the *h*TLR-4 binding site. Within the four simulated models, the epitope vaccine showed more profound movement for its carboxy end and vicinal regions as compared to that of its *N*-terminus, the thing that allowed proximity of the vaccine *C*-terminus toward the *h*TLR-4 lateral side and near the *h*TLR-4 1:1 homodimerization interface. The latter dynamic behavior was most recognized at the monomeric *h*TLR-4 states as compared to the dimeric ones, as well as at the non-glycosylated *h*TLR-4 state in relation to that bounded to the higher oligosaccharides. Notably, applying both Rg and SASA analysis for the epitope vaccine within the four simulated models showed comparable findings (Supplementary Figure 5). Both analytical parameters showed high values at the beginning of the MD simulation runs, yet as the simulation proceeded, the epitope vaccine attained lower as well as much steady plateaued trajectories till the end of the MD timeframes. It is worth mentioning that higher Rg fluctuations tones (around 15-65 ns), as well as late equilibration plateau (not before 70 ns), were achieved for the non-glucosylated/monomeric states as compared to the glycosylated/dimeric ones (Supplementary Figure 5A). Similar findings were also illustrated with SASA analysis where at non-glucosylated/monomeric states, the epitope vaccine exhibited higher fluctuations around 40 ns and till the end of the MD runs (Supplementary Figure 5B).

The designed epitope vaccine across the simulated models showed significant free binding energies toward the *h*TLR-4 binding sites being estimated as kJ/mol ± SD (Table 4). The highest negative values of binding-free energies were assigned to the dimeric states of *h*TLR-4 rather than their respective monomeric ones. Additionally, the glycosyl-bound target proteins depicted a higher affinity toward the anchored epitope vaccine than those being non-glycosylated. Dissecting the obtained total free binding energies Δ*G*_*Total binding*_ showed a preferential energy contribution for the electrostatic non-bonded interactions over the van der Waal potentials. Across the four simulated models, the comparable pattern of energy term contributions was depicted depending on the glycosylation status of the target *h*TLR-4 protein. Differentially higher electrostatic and lower van der Waal values were depicted for the glycosylated target protein states over the monomeric ones. Regarding the solvation energy term contributions, both non-polar and polar solvation energies were of higher negative and positive values, respectively, for the non-glycosylated target proteins over their respective glycosylated forms. The non-polar solvation energy of interaction was monitored across the 100 ns of MD simulation using the SASA-only model calculation for the individual *h*TLR-4, epitope vaccine, as well as their respective combination at each oligomeric/glucosylic state (Supplementary Figure 6). Almost steady solvation energy terms were depicted for the four simulated proteins across the whole MD simulation run. On the other hand, the ligand's solvation energy of interaction showed a significant drop to lower values starting from around 40 ns and till the end of the MD runs achieving the average energy plateau for more than half the simulation timeframes. Steadier solvation energy tones were assigned for the dimeric/glycosylated targets rather than those of the lower simpler states. Notably, the complex solvation energy patterns were significantly impacted *via* the ligand's values and in turn its respective dynamic behavior rather with those of the simulated *h*TLR-4 proteins.

**Table 4:** Free binding energies (Δ*G*_*Total binding*_) and individual energy terms regarding the designed multitope vaccine at *h*TLR-4 target protein binding site.

**Energy (kJ/mol ±SD)**	**Ligand-receptor complex**			
	**Glycosylated dimer**	**Non-glycosylated dimer**	**Glycosylated monomer**	**Non-glycosylated monomer**
Δ*G_*van der Waal*_*	−732.671 ± 72.004	−780.821 ± 82.502	−782.349 ± 115.500	−932.685 ± 23.538
Δ*G_*Electrostatic*_*	−7954.091 ± 79.714	−7798.818 ± 11.828	−7845.389 ± 86.071	−7710.021 ± 97.634
Δ*G_*Solvation*; *Polar*_*	1529.300 ± 11.244	1650.603 ± 18.770	1497.129 ± 39.595	1743.513 ± 12.268
Δ*G_*Solvation*; *SASA*_*	−98.206 ± 13.476	−106.847 ± 9.345	−97.202 ± 16.784	−111.119 ± 24.432
Δ*G_*Total binding*_*	−7255.668 ± 73.478	−7035.883 ± 38.444	−7227.811 ± 20.760	−7010.312 ± 11.335

**Figure 10:**
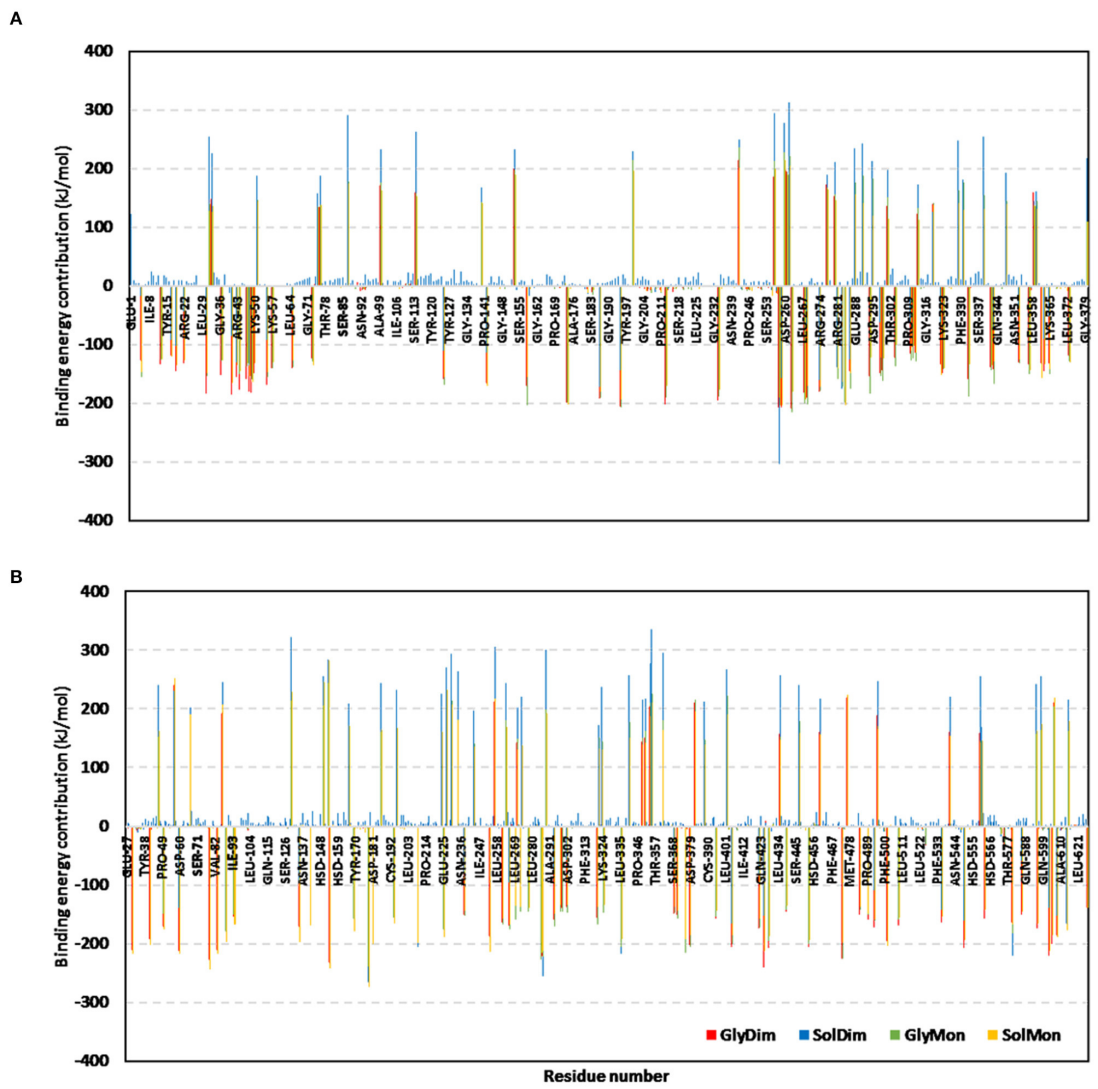
Residue-wise binding-free energy decomposition for the simulated epitope vaccine-*h*TLR-4 complexes. The energy contributions in kJ/mol within the total free binding energies for the residues comprising the simulated **(A)** epitope vaccine; **(B)** *h*TLR-4 target proteins. Energy contributions are represented against the residue number.

The Δ*G*_*Total binding*_ was further decomposed identifying the residue-wise energy contribution for both the vaccine and *h*TLR-4 where the more negative is the better (Figure 10 and Supplementary Figure 7). Findings within the latter figure showed higher positive-valued energy contributions for the vaccines in bound to non-glycosylated *h*TLR-4 states, while the vaccines of glycosylated forms depicted significant negative-valued energy contributions with a wider range of contributing residues. Similar residue-wise energy contribution patterns were assigned for the *h*TLR-4 residues.

### Immune Simulation of the Designed Vaccine

**Figure 11:**
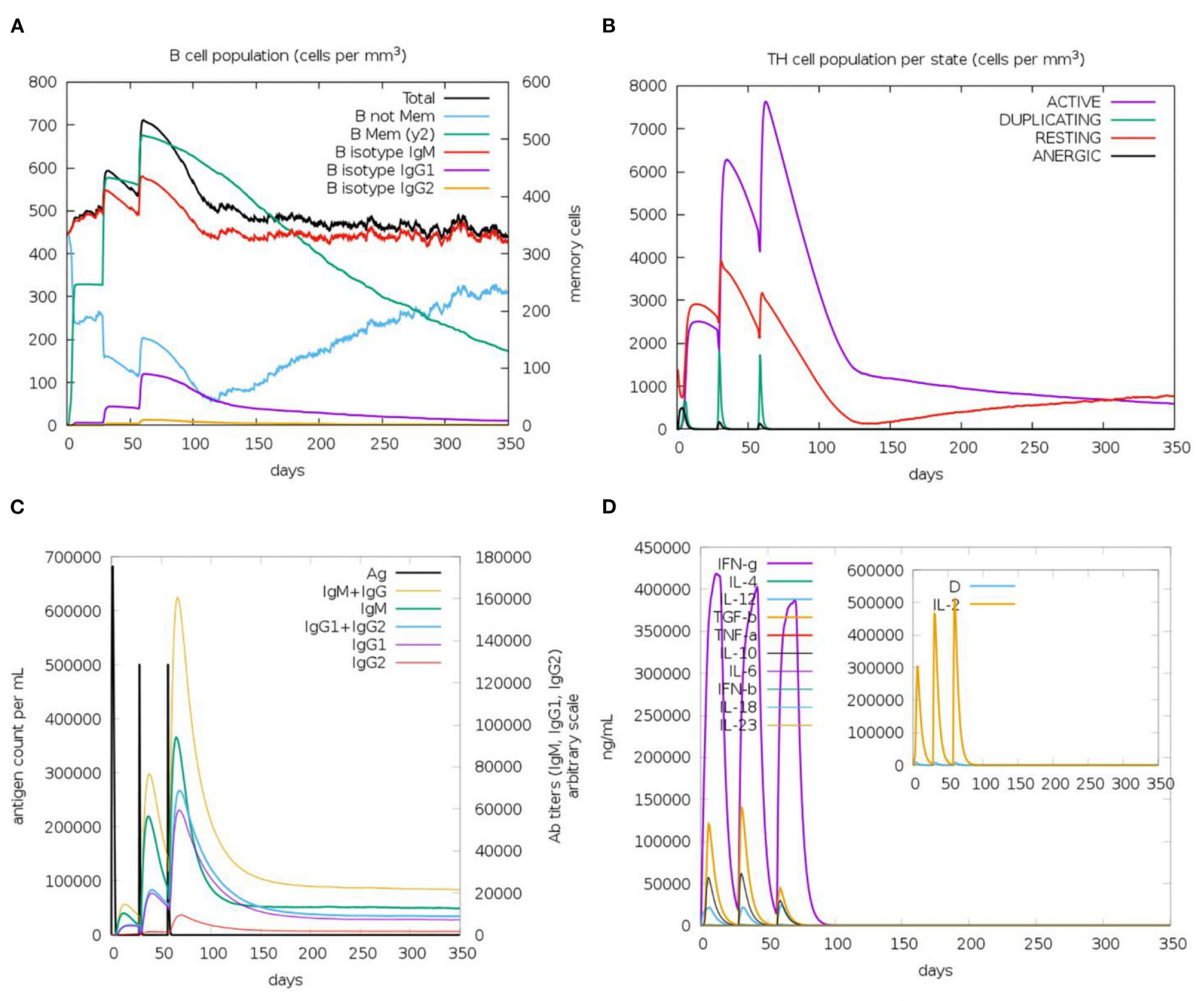
The predicted immune response after designed vaccine injection. **(A,B)** shows the population of B and T cells, respectively, while **(C,D)** demonstrate antibody and cytokine count in a response to vaccine administration.

The immune response regarding antibody titer, cytokines level, B and T cells population is shown in Figure 11. The current study potential vaccine was estimated to stimulate a high level of IgM+IgG which increases with consecutive doses of the vaccine. Regarding cytokines level, several classes were stimulated and INF-γ exhibited the highest level of induced cytokine. Moreover, the count of stimulated T and B cells demonstrated an increase with the doses of the vaccine, and the highest level was obtained as a result of the second booster dose of the vaccine.

## Discussion

Many microorganisms face difficulty in cultivation or attenuation leading to undesirable immune response, proving that the classical approaches for vaccine development against these pathogens require a technical revolution (68). Therefore, the last few years witnessed a large turn in the employed approaches for vaccine development where the multi-omics approaches stepped forward and preceded the traditional ones (69). Recent studies that utilized bioinformatics and structural biology tools for the generation of epitope-based vaccines, that included the antigenic parts only and demonstrated a promising ability for fighting against pathogenic microorganisms, represented a large percentage of the whole studies directed to the vaccine development (70).

The development of an effective vaccine against the several pathotypes of *E. coli* has faced many obstacles. The complex nature of this bacteria and its genetic plasticity hinders the trials for vaccine development. Additionally, lack of broadly applicable testing to assess disease burden, particularly in remote areas where incidence may be quite high besides the occurrence of several bacterial pathotypes are another barriers (71). The current study tried to address these difficulties by analyzing the complete genome of *E. coli* to find common conserved vaccine candidates that cover different pathotypes then assess the characteristics of this potential vaccine computationally before moving to the future phase of wet lab validation. In our analysis, the reverse vaccinology approach was employed to generate a shortlist of potential vaccine candidates after analysis of *E. coli* complete proteome then immunoinformatics computational tools were applied for designing a multitpe vaccine based on filtered vaccine candidates. Only two outer membrane proteins (LptD and BamA) were chosen after applying the filtration steps on 5,155 proteins. The same approach has been reported to be successful in protein filtration and potential vaccine candidature with many pathogens, for instance; *Klebsiella Pneumoniae* (72), *Staphylococcus aureus* (19), *Mycobacterium tuberculosis* (73), *Shigella flexneri* (74), *Pseudomonas aeruginosa* (75), *Moraxella catarrhalis* (76), and Nipah virus (18). In addition to that, *E. coli* was one of the main pathogens that were analyzed for designing a vaccine through a bioinformatics approach in many previous studies. We can divide these studies into two main categories, firstly, studies that just predicted single B and T cell epitopes of protein candidates of certain pathotypes of *E. coli* such as Khan and Kumar (77) and Mehla and Ramana (78). Secondly, more advanced studies that designed and validated a multitope vaccine through a computational approach such as (79), where the vaccine was designed based on Intimin, Stx, Lt, and Cfa proteins and directed against ETEC and EHEC, Another study (80), the vaccine was designed based on IutA and FimH proteins and directed against UPEC, a third study (81), the vaccine was designed based on the bacterial type-3 secretion system and directed against extraintestinal pathogenic *E. coli*. In the current study, we followed the approach of the second group of studies but at the same time, we introduced two unique points that greatly affect the design and the potential application. First, of all, unlike mentioned studies that selected their vaccine candidates based on literature mentioned virulent proteins of specific *E. coli* pathotype, we nominated the vaccine candidates of the current study after a complete filtration of a reference pathogenic *E. coli* strain where the filtration steps represented a basic portion of the study to select proteins that are antigenic, virulent, with a low similarity percentage with human proteins, essential, and conserved throughout various pathotypes of *E. coli*. Consequently, we came up with two novel vaccine candidates (BamA and LptD), and therefore the designed multitope vaccine based on these proteins would have different sequences and characteristics from previously discussed ones. The criteria of protein conservation moved us to the second unique point in the current study which is cross-reactivity. As mentioned, previous trials targeted only specific *E. coli* pathotype in their vaccine design while in this study we targeted essential proteins with a high percentage of conservation in basic *E. coli* pathotypes thus the predicted vaccine would have a potential activity against different *E. coli* pathotypes. Based on what mentioned above, we can choose the steps of common conserved protein candidates selection for several *E. coli* pathotypes and defining the prominent epitopes of these candidates as the most important steps that shaped the novelty of the current potential vaccine construct.

As mentioned above, the process of vaccine design through the immunoinformatics approach has been applied against several pathogens with a common methodology. On the other hand, with the continuous development in computational tools, a recent study (82) proposed a multitope vaccine against COVID-19 through the integration of a deep learning approach for prediction and design. In that study, DeepVacPred was employed to conduct multiple predictions in a time-saving manner. The current study followed the former methodology, especially that the designed vaccines by this common methodology showed promising results during the *in-vivo* validation (80, 83) and as mentioned above the main focus of this study was the development of a potential vaccine that covers most *E. coli* pathotypes and deeply investigate the characteristics of this construct more than developing novel prediction methodologies.

In the current study, several online servers were used to identify potential vaccine candidates against *E. coli* pathotypes where the nominated proteins were found to be highly conserved in the majority of *E. coli* pathotypes. In addition to that, they were outer membrane proteins, essential for bacterial survival, had a high antigenicity score, and were non-homologous to human proteins to ensure their safety in clinical trials. Bacterial outer membrane proteins play an important role in molecule transporting, membrane integrity maintenance, in addition to pathogenesis (84). Moreover, their easy accession to the host immune system gives them the advantage to be highly potential candidates for vaccine development (85). Lipopolysaccharide (LPS) is a major structural component in most Gram-negative bacteria and it is essential for bacterial growth, LptD is one of eight proteins involved in the proper assembly of LPS after its biosynthesis in *E. coli* (86). Regarding BamA, it belongs to the Omp85 family, which is characterized as a major antigenic and immunogenic protein expressed by most Gram-negative pathogenic bacteria (87).

Vaccine construction using mapped epitopes is a sophisticated strategy to trigger an immune response against infectious agents (88). On the other hand, reliance on peptide vaccines for human usage has faced some limitations as single peptide epitopes were found to be not strong enough to stimulate a suitable and prolonged immune response and exhibited low immunogenicity in comparison with live attenuated vaccines. Additionally, there are some questions about the stability of these peptides to induce the immune response before getting lysed by human proteolytic enzymes (89). To overcome these limitations, the current study proposed vaccine was constructed based on a combination of peptides with suitable linkers where those peptides were filtered through several criteria, and only conserved, highly antigenic, non-allergen, and non-toxic epitopes were selected to design the multitope vaccine. multi-epitope vaccines are considered a better choice than monovalent ones as they can stimulate efficient humoral as well as cellular immune responses (90). In addition to these epitopes, other components were added to the final vaccine construct to improve the immune response for this potential vaccine. β-defensin adjuvant was incorporated to create a deposit of the antigenic compound at the site of the vaccine that is steadily released over time, elongating the robust immune response and overcoming one of the main peptide vaccine limitations (91). Moreover, suitable linkers were used to join the selected epitopes from each candidate protein where they provide effective separation between the epitopes (92). Firstly, EAAAK was employed to improve the bi-functional catalytic activity, give stiffness in addition to enhancing fusion protein stability (93). The second linker, GPGPG, was selected for its ability to induce HTL immune response and the ability to break the junctional immunogenicity, resulting in individual epitopes' restoration of immunogenicity (94). The final linker, KK, was employed because of its ability to bring the pH value close to the physiological range (95). In addition to the epitopes, linkers, and adjuvant, the PADRE sequence was also added as it has been revealed that this sequence could minimize the polymorphism of HLA molecules in the population (96). Analyzing the results of the multitope vaccine assessment showed that, the current potential vaccine construct is antigenic with an antigenicity score of 1.07 which was more than the corresponding scores of the designed multitope vaccines against severe acute respiratory syndrome coronavirus 2 (97), Marburg virus (94), and *Leptospira* (98). Regarding the population coverage, the world coverage showed 100% for the constructed vaccine and that was similar to the corresponding coverage reported in (99). For the binding affinity of the single CTLs to the representative alleles, the scores ranged between −7.6 and −8.4, which was smaller than the range reported in (100) but at the same time was large enough when compared to the control binding score of the current study. Moving to the binding energy of the whole multitope vaccine, we reported here a score of −1420.9 kcal/mol, which was smaller than corresponding scores of vaccines predicted with a similar approach (18, 69, 76), implying that a strong binding with TLR would occur. Collectively, The final construct exhibited promising physicochemical, immunological, and chemical characteristics when assessed computationally where molecular dynamics simulation studies were adopted to give a close view of the behavior of this potential vaccine with the receptors of the immune system.

Finally, the designed epitope vaccine showed significant confinement and stability within the hTLR-4 binding site throughout the conducted 100 ns explicit molecular dynamic runs. With preferentiality for the dimeric glycosylated target protein, the designed vaccine exhibited steady conventional thermodynamic behavior with *C*α-RMSDs leveling up for more than 70 ns. The adopted *C*α-RMSD analytical tool allows the estimation of molecular deviation from the designated original/reference structure, the thing that can be used for ensuring ligand-target stability/confinement as well as the validity of the MD protocol (101). Obtaining *C*α-RMSD at low values as well as being rapidly equilibrated has been correlated with the strong affinity of the designed vaccine ligand against target protein as well as the successful convergence of the simulated models requiring no further molecular dynamics simulation runs (102). This *C*α-RMSD-based vaccine-*h*TLR-4 stability was comparable to the stability of several reported proteinaceous multitope vaccines targeting different microorganisms TLRs (103, 104). Regarding the obtained residue-wise fluctuation analysis, the higher *C*α-RMSF values of the epitope vaccine as compared to *h*TLR-4 can be reasonably correlated to their differential tertiary structure folding and/or packing. Generally, the *C*α-RMSF flexibility analysis tool estimates the averaged deviations of protein's residues in relation to their reference positions, the thing that would provide a valuable evaluation of protein's residues regarding their respective dynamic behavior being represented through flexibility and fluctuation (105). In these regards, the incorporation of long α-helices with flexible β-loop connections within the vaccine's designed structures would rationalize the initial relaxation and significant convergence into more stable compacted conformations. On the other hand, *h*TLR-4 exhibited densely packed shoe-like architecture with plenty of highly ordered parallel β-sheets. This differential inherited flexibility was also highlighted through the 3D-conformational analysis between initial and last frames as well as the adopted dihedral coordinate-based normal mode analysis. Having non-uniform stiffness/immobility profiles, as well as high B-factor, deformability, and mobility indices, conferred the profound flexibility being assigned for the vaccine (50). The assigned high immobility profiles for the epitope vaccine were most recognized through its carboxy-terminal amino acids and vicinal residues as being clearly demonstrated through the conformational analysis as well as RMSF, Rg, and SASA findings.

It is worth mentioning that the high RMSF flexibility of the 301–380 residue range was highly reasoned since these residues started the MD simulation being apart from the *h*TLR-4 interface and then ended being near the hTLR-4 lateral side. Nevertheless, the epitope vaccine rapidly attained a more stable compacted conformation/orientation in relation to the bound *h*TLR-4 as the MD runs proceeded. The latter more profoundly stable conformation/orientation of the vaccine was mostly related to the movement of this residue range toward *h*TLR-4 lateral side representing the dimerization interface. This was confirmed through the Rg and SASA analysis where values significantly dropped as the MD simulation proceeded reaching to lower steady trajectories for more than half of the MD simulation timeframes. Generally, Rg accounts for the global stability of either ligand/protein ternary structures, where such stability parameter is defined as the mass-weighted RMSD for atom groups in relation to their respective common center of mass (106). Thus, the depicted dynamic behavior of simulated vaccine to exhbit low Rgs maintaining a plateau around an average value conferred significant stability/compactness at the *h*TLR-4 binding. Notably, the Rg finding further highlighted the preferential vaccine anchoring at the dimeric and/or glycosylated *h*TLR-4 states since the monomeric and/or non-glycosylated ones achieved higher fluctuations and late Rg equilibration trajectories (not before 70ns) suggesting non-optimal compactness and intermolecular binding around these timeframes. Findings from the ligand's SASA analysis came in good agreement with the above preferential complex stability since the simulated vaccine showed steadier SASA tone along the average equilibration plateau around 40 ns and till the simulation end. Since SASA is a quantitative measurement about the extent of protein/solvent interaction correlating for molecular surface area assessable to solvent, thus, low SASA tones imply relative structural shrinkage under the impact of the solvent surface charges yielding more compact and stable conformations (107). The latter findings were also consistent with the vaccine's non-polar solvation energy (only SASA-model) across the MD simulation runs. It is worth mentioning that all above epitope-*h*TLR-4 flexibility patterns were also similarly depicted within several reported studies investigating the potential binding affinity of peptide-based vaccines toward microbial TLRs (103, 104).

The presented study further highlights the impact of *h*TLR-4 oligomerization as well as oligosaccharide states on vaccine binding. Depicting lower *C*α-RMSD and RMSF values with more steady tones at glycosylated dimeric model raised the suggestion that *N*-glycosylation and *h*TLR-4 dimerization were beneficial for vaccine anchoring at the target binding site. Accumulated evidence has illustrated the importance of TLR ectodomains' *N*-glycosylation for orchestrating the localization and signaling capacity (108). Additionally, *N*-linked glycosylation (sialylation) of *h*TLR-4 and its coreceptor, MD-2, enhances the lipoprotein-driven nuclear factor kappa-B activation, cytokine expressions, and, as well as regulates *h*TLR-2 and *h*TLR-3 signaling pathway (109–111). Furthermore, sialylated residues are important for mediating the association between *h*TLR-4 and MD-2, enhancing *h*TLR-4 dimerization, as well as the assembly of complete TLRs signaling complexes (108, 112). Thus, having the importance in enhancing ligand anchoring at TLRs' binding sites as well as facilitating TLRs dimerization it was highly reasoned why highly stabilized/steady thermodynamic behaviors, as well as less fluctuating/, mobilized residues were assigned for both glycosylated dimeric *h*TLR-4/vaccine complex. The MM/PBSA-driven free binding energy calculations also highlighted the higher affinity of the simulated vaccine toward the glycosylated *h*TLR-4 in relation to those being non-glycosylated. Depicting higher negative total free binding energies as well as more pronounced Coloumb's electrostatic energy contributions were highlighted for the more favored vaccine anchoring/affinity toward the glycosylated target proteins. Binding to the *N*-glycosylation chains was also found satisfactory to counterbalance the predicted electrostatic penalties and solvation energies during epitope vaccine ligand binding since lower polar solvation energy terms (Δ*G*_Solvation_) were depicted at the glycosylated models. This was also confirmed through monitoring the non-polar solvation energy *via* the only SASA-model across the MD simulation runs.

Generally, solvation energy terms represent significant repulsive forces against the ligand-binding since binding is a solvent displacement process. It was depicted that these large repulsive forces were mediated majorly by the *h*TLR-4 residues rather than by the vaccine amino acids as being depicted within the residue-wise energy contributions the thing that could be related to the high ordered water molecules at the hydrophobic surface of the TLR-2 ligand-binding site. Thus, the presence of *N*-linked glycosylation chains would minimize such repulsive penalties against the vaccine binding. On the other hand, the total non-polar interactions (Δ*G*_van der Waal_ plus Δ*G*_SASA_) were shown to be higher at the non-glycosylated models conferring their respective larger surface area as well as higher hydrophobic potentialities toward vaccine binding. The latter was rationalized since accumulated evidence has considered the general TLRs binding site to be extended and more hydrophobic in nature (52–55, 113–115). Based on the above evidence, it was satisfactory that the designed epitope vaccine depicted significant binding affinity toward the *h*TLR-4 binding pocket with higher preferentiality toward the glycosylated dimeric state.

## Conclusion

The current study aimed to demonstrate the role of modern approaches for vaccine development as a potential solution to fight resistant pathogens. Here, we reported two proteins namely BamA and LptD, after the filtration of the whole proteome of *E. coli* reference strain, to act as a base for multitope vaccine construct against *E. coli* pathotypes. The multitope construct included top-ranked epitopes of the filtered proteins in addition to beta-defensin and PADRE peptide. The molecular modeling simulation studies illustrated relevant affinity/binding of the designed epitope vaccine toward the *h*TLR-4 binding pocket, yet with higher preferentiality toward the glycosylated dimeric state. Finally, The predicted physicochemical and immunological characteristics of the constructed vaccine nominated it as a potential solution against several *E. coli* pathotypes and recommended its movement to wet lab validation.

## Data Availability Statement

The datasets presented in this study can be found in online repositories. The names of the repository/repositories and accession number(s) can be found in the article/Supplementary Material.

## Author Contributions

MS, MB, MSA, MA, and KD: conceptualization, methodology, and original draft preparation. SA, EF, SE, AL, and RE: writing—review and editing. MS and MA: supervision and project administration. All authors contributed to the article and approved the submitted version.

## Funding

This research was funded by Taif University Researchers Supporting Project number (TURSP-2020/202), Taif University, Taif, Saudi Arabia.

## Conflict of Interest

The authors declare that the research was conducted in the absence of any commercial or financial relationships that could be construed as a potential conflict of interest.

## Publisher's Note

All claims expressed in this article are solely those of the authors and do not necessarily represent those of their affiliated organizations, or those of the publisher, the editors and the reviewers. Any product that may be evaluated in this article, or claim that may be made by its manufacturer, is not guaranteed or endorsed by the publisher.

## Supplementary Material

Supplementary Figure 1 Predicted positions of MHC-I peptides (yellow color) in the 3D structure of HLA-B^*^44:03 receptor (red color), structures **(A–F)** are for epitopes number 1, 2, 3, 4, 5, and 6, respectively, from Supplementary Table 3.

Supplementary Figure 2 Predicted positions of MHC-II peptides (yellow color) in the 3D structure of HLA-DRB1^*^04:01 receptor (red color), structures **(A–F)** are for epitopes number 1, 2, 3, 4, 5, and 6, respectively, from Supplementary Table 3.

Supplementary Figure 3 Graphical map for the designed multitope vaccine.

Supplementary Figure 4 The 3D model of the 9 predicted conformational B-cell epitopes in the refined final vaccine construct where the yellow residues represent the epitopes and the gray ones are for the rest of the predicted vaccine. Letters from A to I represents the predicted epitopes in Supplementary Table 7 from 1 to 9, respectively.

Supplementary Figure 5 Global stability of the epitope vaccine at the four simulated models across 100 ns explicit molecular dynamics simulation runs. Time-evolution of **(A)** Rg, **(B)** SASA, along molecular dynamics timeframes (ns).

Supplementary Figure 6 Non-polar solvation energy terms using the SASA-only model calculation for the four investigated epitope vaccine ligand-bound hTLR-4 target models across 100 ns explicit molecular dynamics simulation runs. Time-evolution of **(A)** Proteins, **(B)** Ligands, and **(C)** Complexes, along molecular dynamics timeframes (ns).

Supplementary Figure 7 Residue-wise binding-free energy decomposition for the simulated epitope vaccine-hTLR-4 complexes. 3D-representation (Cartoon) for the regions implying favored binding interactions on the initial/reference vaccine/hTLR-4 complex. Glycosylated dimeric, non-glycosylated dimeric, glycosylated monomeric, and non-glycosylated monomeric states are at upper-left, upper-right, lower-left, and lower right quadrant, respectively (Only one protomer is shown for clarity). Regions of the proteins are in spectrum colors from dark blue (negative-valued ΔG kJ/mol conferring highly favored attractive binding forces) down to dark red (positive-valued ΔG kJ/mol conferring unfavored high repulsive binding forces).

Supplementary Table 1 Characteristics of Potential Vaccine Candidates for *E. coli* O157:H7 str. Sakai.

Supplementary Table 2 Protein conservancy of the selected candidates among *E. coli* pathotypes.

Supplementary Table 3 Selected epitopes' binding energies with representative MHC-I and MHC-II alleles.

Supplementary Table 4 Reacting alleles and population coverage of filtered CLTs and HTLs.

Supplementary Table 5 Population coverage percentage of the multitope vaccine and its constructing peptides (CTLs and HTLs).

Supplementary Table 6 Predicted physicochemical properties of the chimeric vaccine.

Supplementary Table 7 The predicted conformational B cell epitopes from the refined vaccine 3D construct.
