# Supplementary figures and images for: In silico Designing of an Epitope-Based Vaccine Against Common E. coli Pathotypes
Source: Front Med (Lausanne). 2022 Mar 4;9:829467. doi: 10.3389/fmed.2022.829467 (PMC8931290; doi:10.3389/fmed.2022.829467)

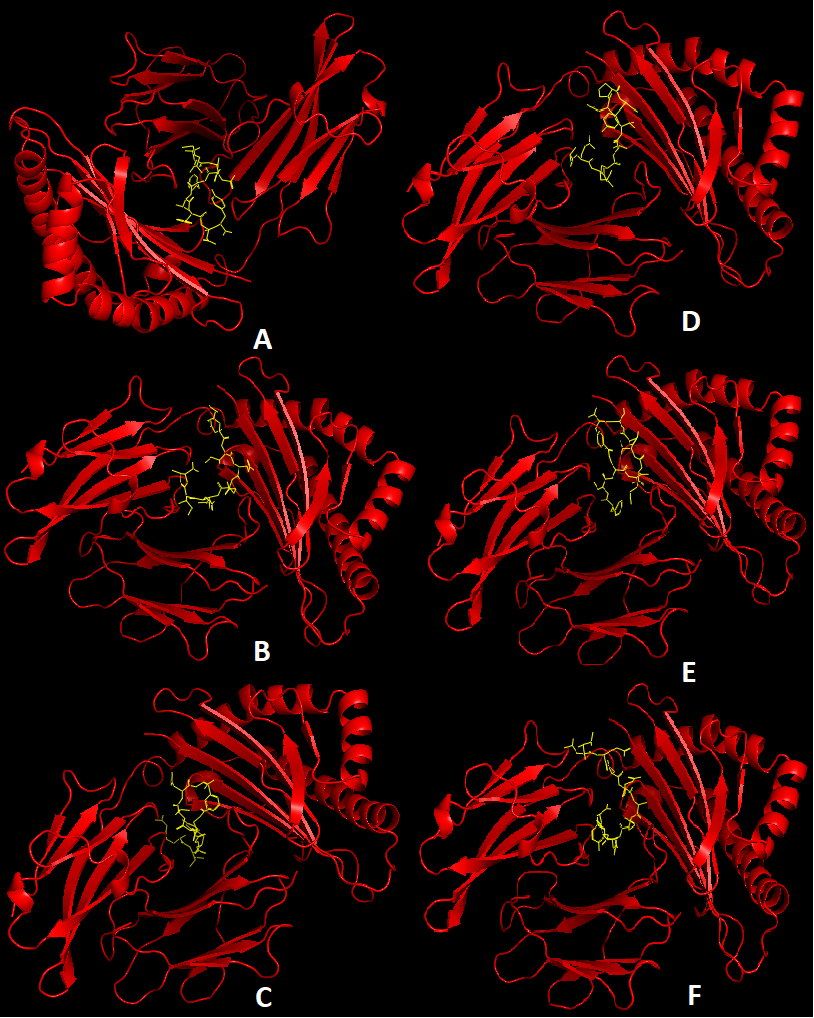

Supplement: Supplementary Figure 1 — Predicted positions of MHC-I peptides (yellow color) in the 3D structure of HLA-B*44:03 receptor (red color), structures (A–F) are for epitopes number 1, 2, 3, 4, 5, and 6, respectively, from Supplementary Table 3. [file Image_1.PNG]

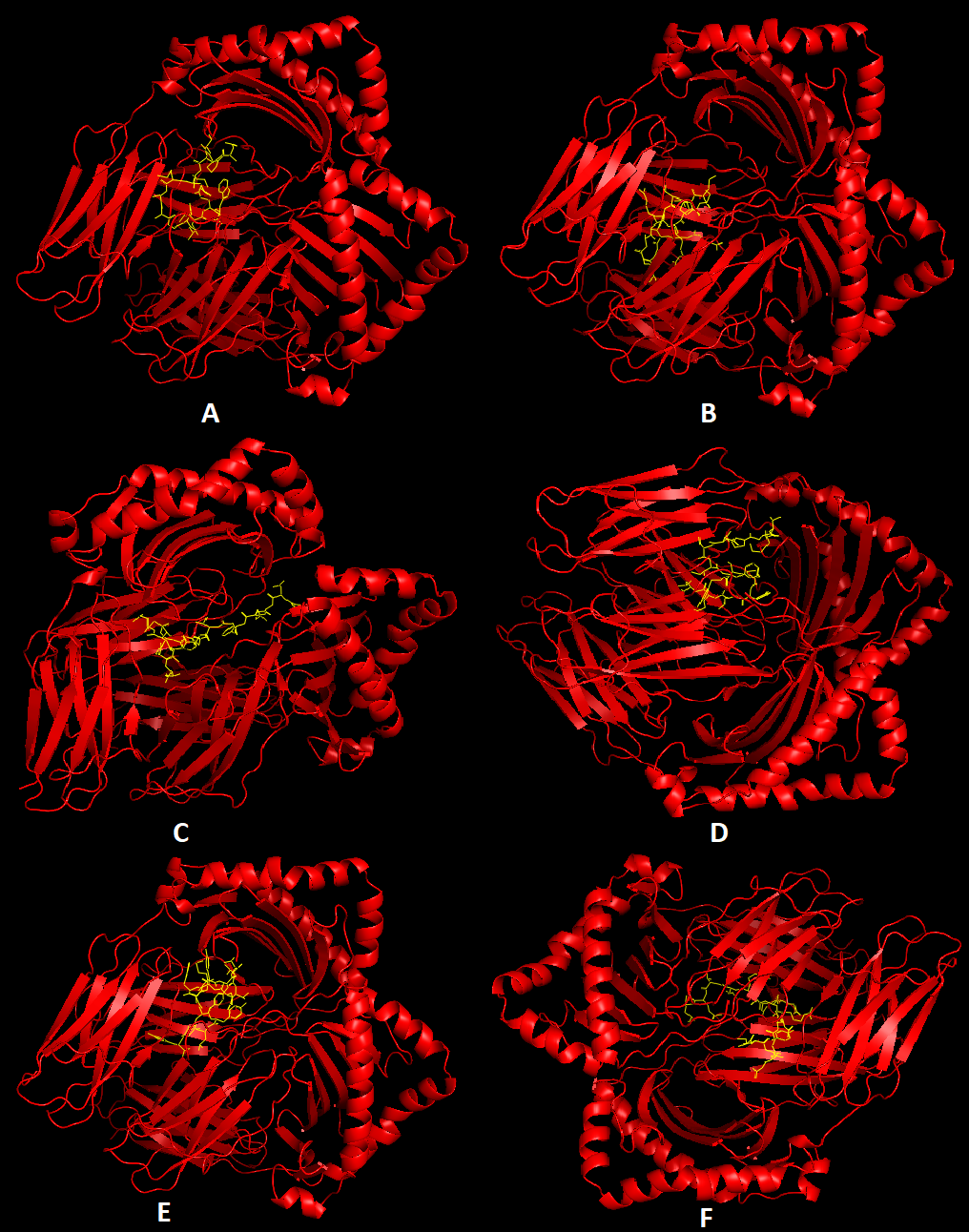

Supplement: Supplementary Figure 2 — Predicted positions of MHC-II peptides (yellow color) in the 3D structure of HLA-DRB1*04:01 receptor (red color), structures (A–F) are for epitopes number 1, 2, 3, 4, 5, and 6, respectively, from Supplementary Table 3. [file Image_2.PNG]

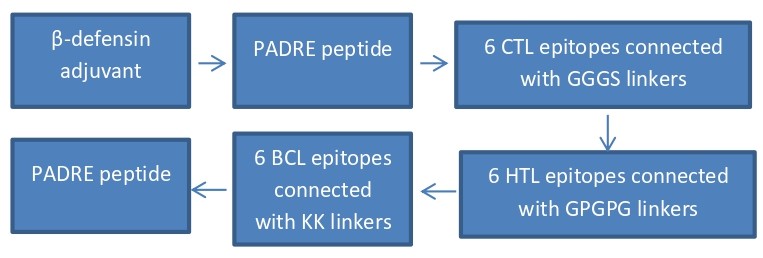

Supplement: Supplementary Figure 3 — Graphical map for the designed multitope vaccine. [file Image_3.JPEG]

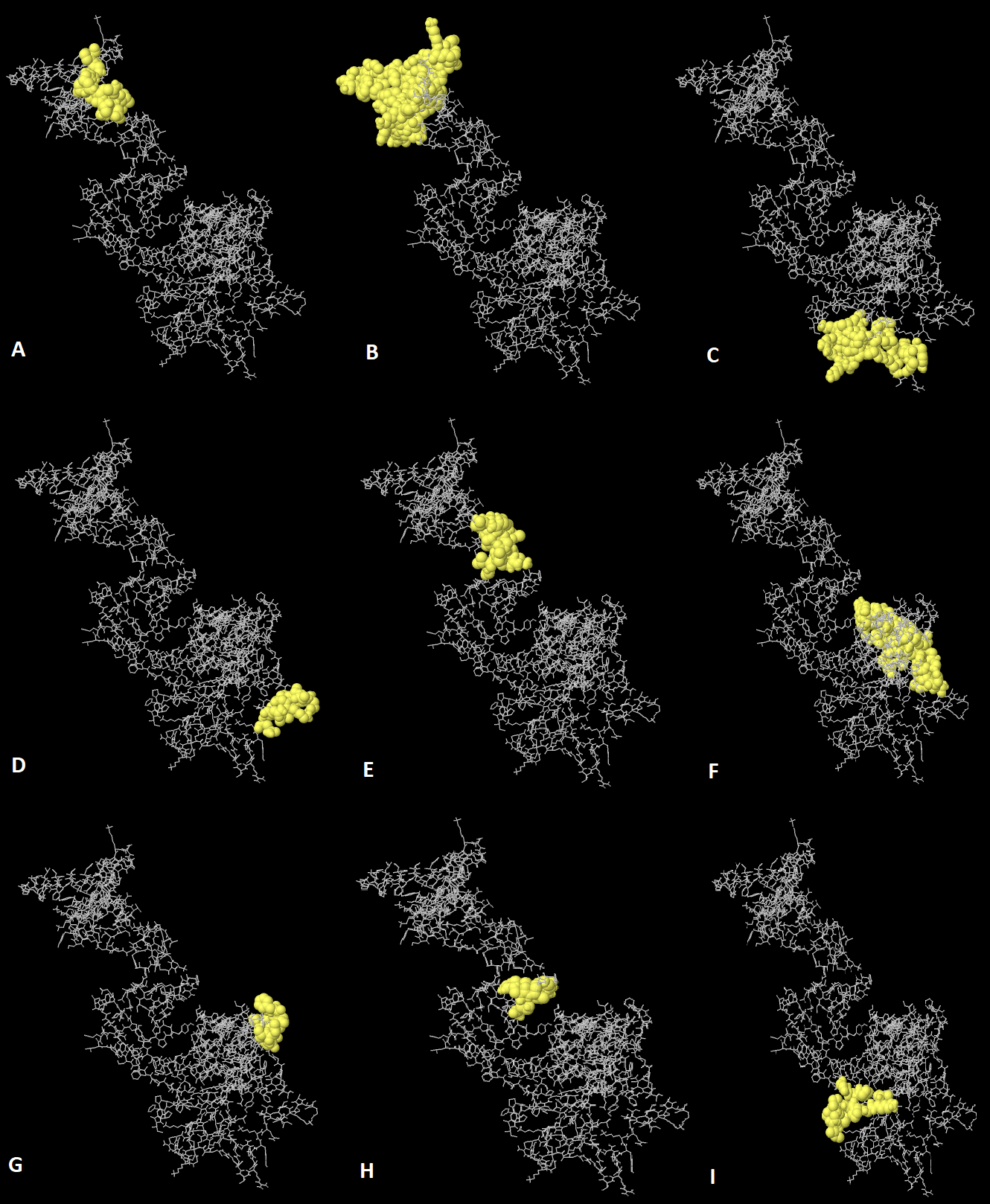

Supplement: Supplementary Figure 4 — The 3D model of the 9 predicted conformational B-cell epitopes in the refined final vaccine construct where the yellow residues represent the epitopes and the gray ones are for the rest of the predicted vaccine. Letters from A to I represents the predicted epitopes in Supplementary Table 7 from 1 to 9, respectively. [file Image_4.PNG]

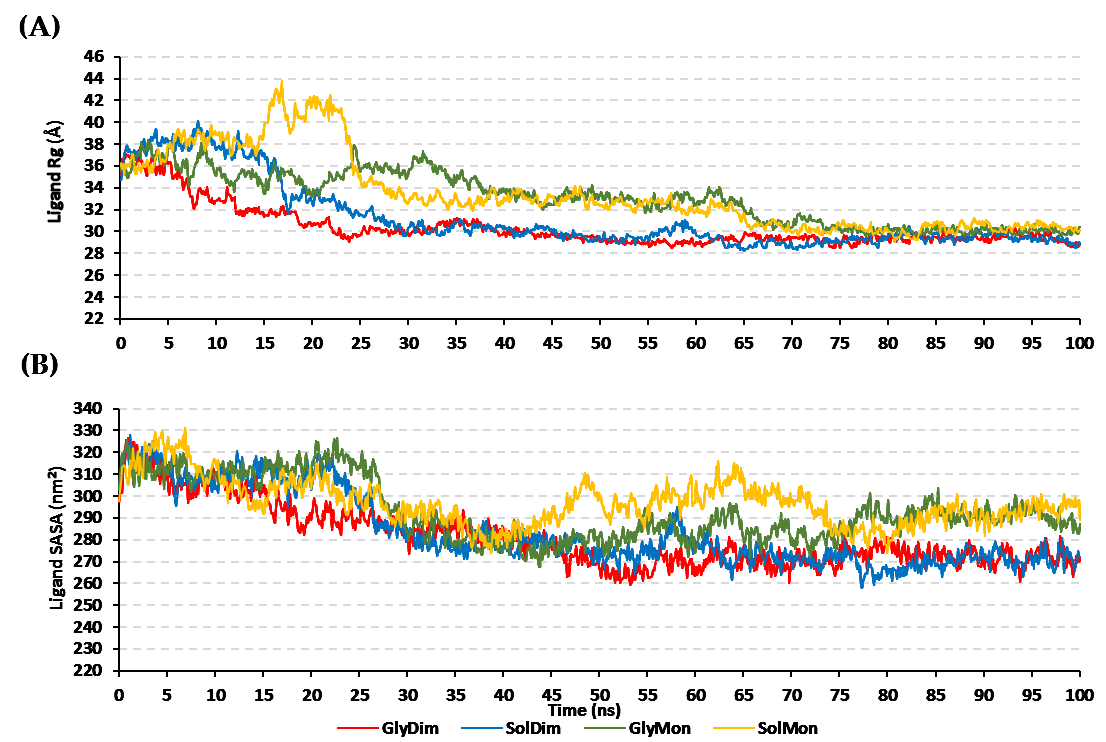

Supplement: Supplementary Figure 5 — Global stability of the epitope vaccine at the four simulated models across 100 ns explicit molecular dynamics simulation runs. Time-evolution of (A) Rg, (B) SASA, along molecular dynamics timeframes (ns). [file Image_5.PNG]

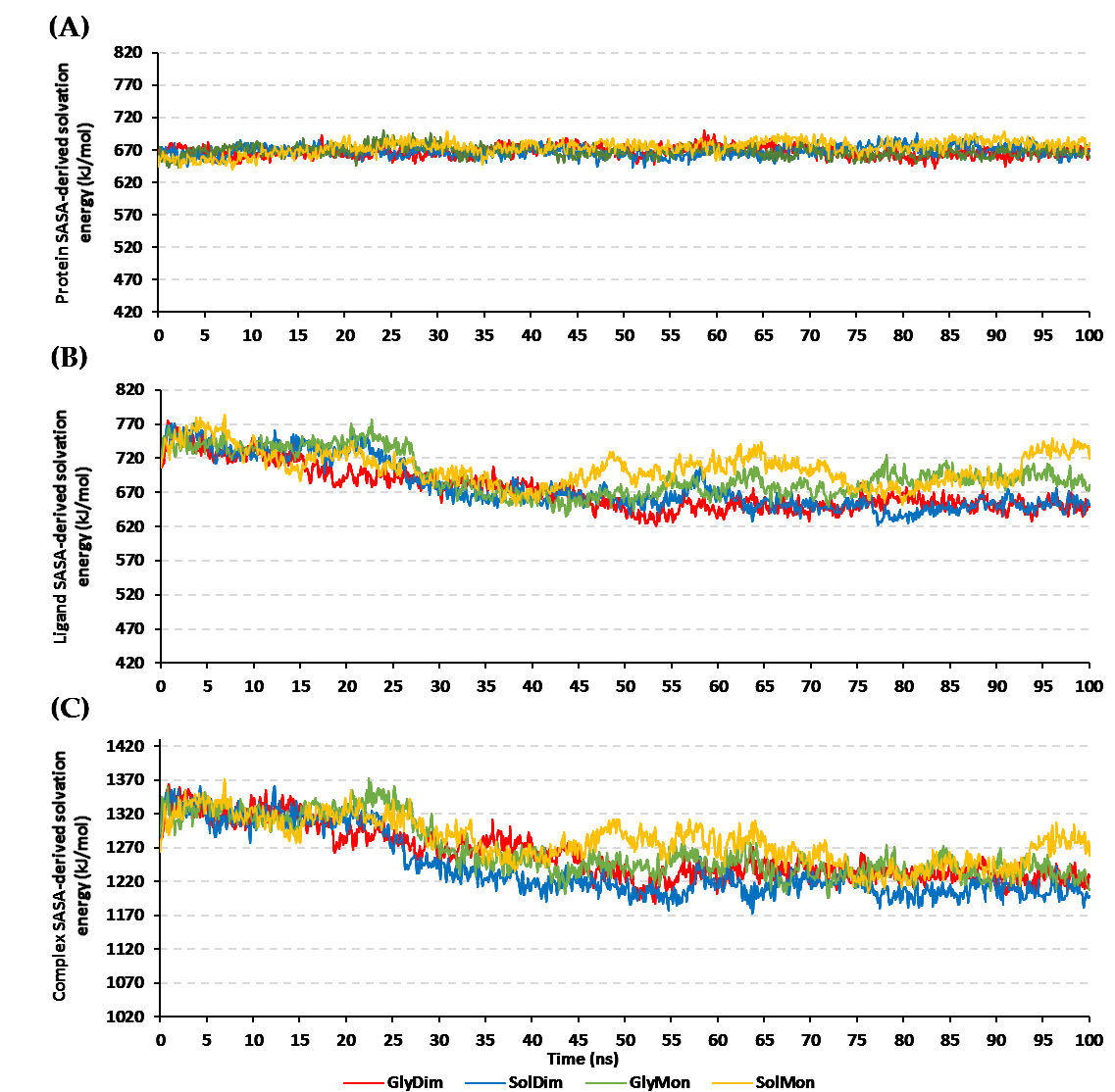

Supplement: Supplementary Figure 6 — Non-polar solvation energy terms using the SASA-only model calculation for the four investigated epitope vaccine ligand-bound hTLR-4 target models across 100 ns explicit molecular dynamics simulation runs. Time-evolution of (A) Proteins, (B) Ligands, and (C) Complexes, along molecular dynamics timeframes (ns). [file Image_6.PNG]

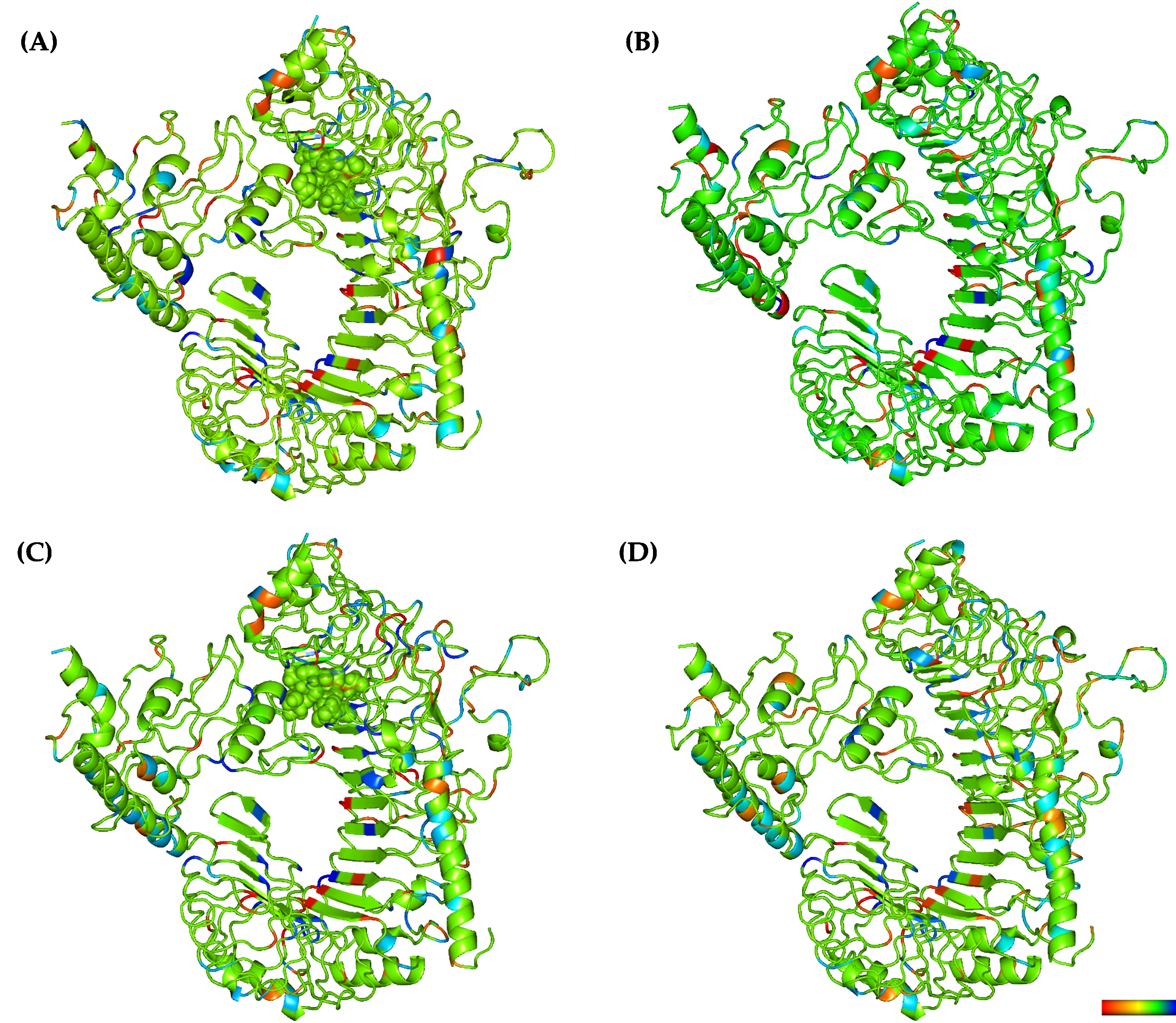

Supplement: Supplementary Figure 7 — Residue-wise binding-free energy decomposition for the simulated epitope vaccine-hTLR-4 complexes. 3D-representation (Cartoon) for the regions implying favored binding interactions on the initial/reference vaccine/hTLR-4 complex. Glycosylated dimeric, non-glycosylated dimeric, glycosylated monomeric, and non-glycosylated monomeric states are at upper-left, upper-right, lower-left, and lower right quadrant, respectively (Only one protomer is shown for clarity). Regions of the proteins are in spectrum colors from dark blue (negative-valued ΔG kJ/mol conferring highly favored attractive binding forces) down to dark red (positive-valued ΔG kJ/mol conferring unfavored high repulsive binding forces). [file Image_7.PNG]
