# Supplementary material for: In silico Designing of an Epitope-Based Vaccine Against Common E. coli Pathotypes
Source: Front Med (Lausanne). 2022 Mar 4;9:829467. doi: 10.3389/fmed.2022.829467 (PMC8931290; doi:10.3389/fmed.2022.829467)
Supplement: Supplementary Table 1 — Characteristics of Potential Vaccine Candidates for E. coli O157:H7 str. Sakai. [file Table_1.DOCX]

**Supplementary table 1**. Characteristics of Potential Vaccine Candidates for *E. coli* O157:H7 str. Sakai.

| **Protein name** | **PSORb**  **result** | **Essential** | | **Virulent** | **Non-human homolog** | **Molecular weight (KDa)** | **Transmembrane**  **helices** | **Antigenicity**  **score** |
| --- | --- | --- | --- | --- | --- | --- | --- | --- |
| **BamA** | Outer membrane | | √ | √ | √ | 90.55 | 0 | 0.57 |
| **LptD** | Outer membrane | | √ | √ | √ | 89.66 | 0 | 0.59 |
