# Supplementary material for: In silico Designing of an Epitope-Based Vaccine Against Common E. coli Pathotypes
Source: Front Med (Lausanne). 2022 Mar 4;9:829467. doi: 10.3389/fmed.2022.829467 (PMC8931290; doi:10.3389/fmed.2022.829467)
Supplement: Supplementary Table 2 — Protein conservancy of the selected candidates among E. coli pathotypes. [file Table_2.DOCX]

Supplementary table 2. Protein conservancy of the selected candidates among *E. coli* pathotypes.

| ***E. coli* pathotype** | **Representative serotype** | **Amino acid sequence homology percentage (%)** | |
| --- | --- | --- | --- |
|  |  | **BamA** | **LptD** |
| UPEC | O1 | 100 | 99.62 |
| EHEC | O157:H7 | 100 | 100 |
| EPEC | O86:H34 | 99.88 | 98.85 |
| EIEC | O144 | 100 | 99.74 |
| EAEC | O44:H18 | 100 | 99.62 |
| ETEC | O6:H16 | 100 | 99.62 |
| DAEC | O2:H6 | 100 | 98.85 |
| AIEC | O6 | 100 | 99.74 |
| STEAEC | O26 | 100 | 99.74 |
| NMEC | O18:H7 | 100 | 99.74 |
