# Supplementary material for: In silico Designing of an Epitope-Based Vaccine Against Common E. coli Pathotypes
Source: Front Med (Lausanne). 2022 Mar 4;9:829467. doi: 10.3389/fmed.2022.829467 (PMC8931290; doi:10.3389/fmed.2022.829467)
Supplement: Supplementary Table 3 — Selected epitopes' binding energies with representative MHC-I and MHC-II alleles. [file Table_3.DOCX]

**Supplementary table3.** Selected epitopes’ binding energies with representative MHC-I and MHC-II alleles.

| No. | Epitope | MHC-I allele | Binding energy  (kcal/mol) | Epitope | MHC-II allele | Binding energy  (kcal/mol) |
| --- | --- | --- | --- | --- | --- | --- |
| 1 | KTDDFTFNY |  | -8.4 | DPSNIRMSAGIALQW |  | -7.4 |
| 2 | AELSVTNPY |  | -7.7 | QRVAVGAALLSMPVR |  | -7.1 |
| 3 | AEIQQINIV | HLA-B*44:03 | -7.6 | TNKSYGTDVTLGFPI | HLA-DRB1*04:01 | -8.1 |
| 4 | FSEQNTSSY |  | -7.9 | KVGPVSIFYSPYLQL |  | -7.8 |
| 5 | RIYGQAVHF |  | -8.5 | AKYTTTNYFEFYLPY |  | -7.4 |
| 6 | TLEPRAQYLY |  | -7.8 | SSIEYRRDEDRLVQL |  | -7.8 |
