# Supplementary material for: In silico Designing of an Epitope-Based Vaccine Against Common E. coli Pathotypes
Source: Front Med (Lausanne). 2022 Mar 4;9:829467. doi: 10.3389/fmed.2022.829467 (PMC8931290; doi:10.3389/fmed.2022.829467)
Supplement: Supplementary Table 4 — Reacting alleles and population coverage of filtered CLTs and HTLs. [file Table_4.DOCX]

| **Peptide** | **Reacting alleles** | **World coverage percentage** |
| --- | --- | --- |
| KTDDFTFNY | HLA-A*01:01, HLA-A*30:02, HLA-B*58:01, HLA-A*11:01, HLA-A*32:01, HLA-B*57:01, HLA-A*26:01, HLA-B*35:01, HLA-B*53:01, HLA-B*15:01, HLA-A*68:01, HLA-A*30:01, HLA-A*31:01, HLA-B*44:03, HLA-A*33:01, HLA-B*44:02, HLA-A*02:06, HLA-A*23:01, HLA-A*24:02, HLA-A*03:01 | 87.85% |
| AELSVTNPY | HLA-B*44:03, HLA-A*01:01, HLA-B*15:01, HLA-B*40:01, HLA-B*44:02, HLA-A*30:02, HLA-A*26:01, HLA-B*35:01, HLA-B*53:01, HLA-A*32:01, HLA-A*68:01, HLA-A*23:01, HLA-B*58:01, HLA-A*24:02 | 72.86% |
| AEIQQINIV | HLA-B*40:01, HLA-B*44:03, HLA-B*44:02, HLA-A*02:06, HLA-A*68:02, HLA-B*15:01 | 31.55% |
| FSEQNTSSY | HLA-B*35:01, HLA-A*01:01, HLA-A*26:01, HLA-B*15:01, HLA-B*58:01, HLA-B*57:01, HLA-A*68:01, HLA-A*24:02, HLA-A*23:01, HLA-A*30:02 | 62.65% |
| RIYGQAVHF | HLA-A*32:01, HLA-B*15:01, HLA-B*58:01, HLA-B*57:01, HLA-A*30:02, HLA-A*23:01, HLA-A*02:03, HLA-A*30:01, HLA-A*24:02, HLA-A*26:01, HLA-A*02:06, HLA-A*02:01, HLA-A*03:01, HLA-B*35:01, HLA-B*53:01, HLA-A*31:01, HLA-A*11:01, HLA-B*07:02, HLA-B*08:01, HLA-A*68:01, HLA-A*33:01, HLA-B*51:01, HLA-A*68:02, HLA-B*44:03 | 96.09% |
| TLEPRAQYLY | HLA-A*01:01, HLA-A*30:02, HLA-B*44:03, HLA-B*44:02, HLA-A*26:01, HLA-B*15:01, HLA-B*35:01, HLA-A*32:01, HLA-A*03:01, HLA-A*11:01, HLA-A*68:01, HLA-B*53:01 | 71.25% |
| DPSNIRMSAGIALQW | HLA-DRB1*09:01, HLA-DPA1*02:01, HLA-DPB1*14:01, HLA-DRB1*07:01, HLA-DRB3*02:02, HLA-DRB1*13:02, HLA-DQA1*01:02, HLA-DQB1*06:02, HLA-DRB1*01:01, HLA-DQA1*05:01, HLA-DQB1*03:01, HLA-DRB1*15:01, HLA-DRB1*03:01, HLA-DRB4*01:01, HLA-DRB5*01:01, HLA-DQA1*04:01, HLA-DQB1*04:02, HLA-DRB1*08:02, HLA-DRB3*01:01 | 95.65% |
| QRVAVGAALLSMPVR | HLA-DPA1*02:01, HLA-DPB1*14:01, HLA-DQA1*05:01, HLA-DQB1*03:01, HLA-DRB1*01:01, HLA-DQA1*01:02, HLA-DQB1*06:02, HLA-DRB1*09:01, HLA-DPA1*03:01, HLA-DPB1*04:02 | 89.97% |
| TNKSYGTDVTLGFPI | HLA-DRB3*01:01, HLA-DRB1*04:01, HLA-DRB1*03:01 | 27.97% |
| KVGPVSIFYSPYLQL | HLA-DPB1*04:01, HLA-DPA1*01:03, HLA-DPB1*02:01, HLA-DPA1*03:01, HLA-DPB1*04:02, HLA-DPA1*02:01, HLA-DPB1*01:01, HLA-DRB1*15:01, HLA-DRB1*09:01, HLA-DPB1*05:01, HLA-DRB1*07:01, HLA-DQA1*01:01, HLA-DQB1*05:01, HLA-DPB1*14:01, HLA-DQA1*05:01, HLA-DQB1*02:01, HLA-DRB1*03:01, HLA-DRB1*04:05 | 99.86% |
| AKYTTTNYFEFYLPY | HLA-DPA1*01:03, HLA-DPB1*04:01, HLA-DPA1*02:01, HLA-DPB1*05:01, HLA-DPB1*02:01, HLA-DQA1*01:01, HLA-DQB1*05:01, HLA-DPB1*01:01, HLA-DRB3*01:01, HLA-DQA1*05:01, HLA-DQB1*02:01 | 98.41% |
| SSIEYRRDEDRLVQL | HLA-DRB3*01:01, HLA-DRB1*03:01 | 17.84% |

Supplementary table 4. Reacting alleles and population coverage of filtered CLTs and HTLs.
