# Supplementary material for: In silico Designing of an Epitope-Based Vaccine Against Common E. coli Pathotypes
Source: Front Med (Lausanne). 2022 Mar 4;9:829467. doi: 10.3389/fmed.2022.829467 (PMC8931290; doi:10.3389/fmed.2022.829467)
Supplement: Supplementary Table 5 — Population coverage percentage of the multitope vaccine and its constructing peptides (CTLs and HTLs). [file Table_5.DOCX]

| Peptides | East Asia (%) | South Asia (%) | Europe (%) | East Africa (%) | West Africa (%) | Central Africa (%) | North America (%) | South America (%) | World (%) |
| --- | --- | --- | --- | --- | --- | --- | --- | --- | --- |
| Combined CLTs | 98.18 | 94.73 | 99.68 | 90.78 | 95.49 | 86.04 | 99.06 | 88.3 | 98.55 |
| Combined HLTs | 99.6 | 99.98 | 100 | 100 | 100 | 100 | 100 | 99.99 | 99.96 |
| Multitope vaccine | 99.99 | 100 | 100 | 100 | 100 | 100 | 100 | 100 | 100 |

Supplementary table 5. Population coverage percentage of the multitope vaccine and its constructing peptides (CTLs and HTLs)
