# Supplementary material for: In silico Designing of an Epitope-Based Vaccine Against Common E. coli Pathotypes
Source: Front Med (Lausanne). 2022 Mar 4;9:829467. doi: 10.3389/fmed.2022.829467 (PMC8931290; doi:10.3389/fmed.2022.829467)
Supplement: Supplementary Table 6 — Predicted physicochemical properties of the chimeric vaccine. [file Table_6.DOCX]

**Supplementary table 6.** Predicted physicochemical properties of the chimeric vaccine

| **Physicochemical characteristic** | **Molecular weight** | **Theoretical**  **pI** | **Extinction**  **coefficient** |  | **GRAVY** | **Instability**  **index** | **Aliphatic**  **index** |
| --- | --- | --- | --- | --- | --- | --- | --- |
| Score | 40.33 kDa | 9.66 | 51800 M^-1^ cm^-1^ |  | -0.634 | 34.39 | 56.7 |
