# Supplementary material for: In silico Designing of an Epitope-Based Vaccine Against Common E. coli Pathotypes
Source: Front Med (Lausanne). 2022 Mar 4;9:829467. doi: 10.3389/fmed.2022.829467 (PMC8931290; doi:10.3389/fmed.2022.829467)
Supplement: Supplementary Table 7 — The predicted conformational B cell epitopes from the refined vaccine 3D construct. [file Table_7.DOCX]

| **No.** | **Residues** | **Number of residues** | **Score** |
| --- | --- | --- | --- |
| 1 | _:D295, _:A296, _:V297, _:K298, _:K299, _:T300, _:D301, _:T302, _:Q303, _:R304, _:V305, _:P306, _:G307, _:S308, _:P309 | 15 | 0.84 |
| 2 | _:K310, _:K311, _:K312, _:E313, _:A314, _:P315, _:G316, _:Q317, _:P318, _:E319, _:P320, _:K322, _:K323, _:P324, _:S325, _:Y326, _:F327, _:N328, _:D329, _:F330, _:D331, _:N332, _:K333, _:Y334, _:G335, _:S336, _:S337, _:T338, _:D339, _:G340, _:Y341, _:K342, _:K343, _:Q344, _:T345, _:N346, _:L347, _:D348, _:W349, _:Y350, _:N351, _:S352, _:R353, _:N354, _:T355, _:T356, _:K357, _:L358, _:D359, _:E360, _:S361, _:K362, _:K363, _:A364, _:K365, _:F366, _:V367, _:A368, _:A369, _:W370, _:T371, _:L372, _:K373, _:A374, _:A375, _:A376, _:G377, _:G378, _:G379, _:S380 | 70 | 0.757 |
| 3 | _:Y14, _:Y15, _:C16, _:R19, _:G20, _:G21, _:R22, _:C23, _:A24, _:C28, _:L29, _:K31, _:E32, _:E33, _:Q34, _:I35, _:G36, _:K37, _:C38, _:S39, _:T40, _:R41, _:G42, _:R43, _:K44, _:C45, _:C46, _:R47 | 28 | 0.734 |
| 4 | _:E1, _:A2, _:A3, _:A4, _:K5, _:G6, _:I7, _:I8, _:N9, _:Q12 | 10 | 0.671 |
| 5 | _:G272, _:V273, _:R274, _:V275, _:G276, _:E277, _:S278, _:L279, _:D280, _:R281, _:T282, _:K283, _:K284, _:R286 | 14 | 0.645 |
| 6 | _:F58, _:A61, _:W62, _:L64, _:K65, _:A66, _:A67, _:A68, _:G69, _:G70, _:G71, _:S72, _:K73, _:T74, _:D75, _:D76, _:F79, _:N80, _:Q103, _:N105, _:I106, _:V107, _:G108, _:G110, _:S111, _:F112, _:S113, _:E114, _:Q115, _:N116, _:T117, _:S118, _:S119, _:Y120, _:G121, _:G122, _:G123, _:S124, _:R125, _:I126, _:Y127 | 41 | 0.613 |
| 7 | _:G128, _:Q129, _:A130, _:V131, _:H132, _:F133, _:G134, _:G135, _:G136, _:S137, _:Q144 | 11 | 0.591 |
| 8 | _:G252, _:S253, _:S254, _:I255, _:R258, _:R259 | 6 | 0.522 |
| 9 | _:G204, _:F205, _:P206, _:I207, _:G208, _:P209, _:G210, _:P211, _:G212, _:K213, _:V214, _:P216, _:V217, _:S218 | 14 | 0.514 |

Supplementary table.7: The predicted conformational B cell epitopes from the refined vaccine 3D construct
